# CLEC5A Regulates Japanese Encephalitis Virus-Induced Neuroinflammation and Lethality

**DOI:** 10.1371/journal.ppat.1002655

**Published:** 2012-04-19

**Authors:** Szu-Ting Chen, Ren-Shyan Liu, Ming-Fang Wu, Yi-Ling Lin, Se-Yi Chen, David Tat-Wei Tan, Teh-Ying Chou, I-Shuen Tsai, Lei Li, Shie-Liang Hsieh

**Affiliations:** 1 Department and Institute of Microbiology and Immunology, National Yang-Ming University, Taipei, Taiwan; 2 Genomics Research Center, Academia Sinica, Taipei, Taiwan; 3 Molecular and Genetic Imaging Core, Department of Nuclear Medicine, National Yang-Ming University Medical School and Taipei Veterans General Hospital, Taipei, Taiwan; 4 Institute of Biomedical Sciences, Academia Sinica, Taipei, Taiwan; 5 Department of Neurosurgery Surgical, Taichung Veterans General Hospital, Taichung, Taiwan; 6 Institute of Clinical Medicine, National Yang-Ming University, Taipei, Taiwan; 7 Molecular and Genetic Imaging Core, Department of Biomedical Imaging and Radiological Sciences, National Yang-Ming University, Taipei, Taiwan; 8 Taipei Blood Center, Taiwan Blood Services Foundation, Taipei, Taiwan; 9 Infection and Immunity Research Center, National Yang-Ming University, Taipei, Taiwan; 10 Immunology Center, Taipei Veterans General Hospital, Taipei, Taiwan; Nationwide Children's Hospital, United States of America

## Abstract

CLEC5A/MDL-1, a member of the myeloid C-type lectin family expressed on macrophages and neutrophils, is critical for dengue virus (DV)-induced hemorrhagic fever and shock syndrome in *Stat1* ^−/−^ mice and ConA-treated wild type mice. However, whether CLEC5A is involved in the pathogenesis of viral encephalitis has not yet been investigated. To investigate the role of CLEC5A to regulate JEV-induced neuroinflammation, antagonistic anti-CLEC5A mAb and CLEC5A-deficient mice were generated. We find that Japanese encephalitis virus (JEV) directly interacts with CLEC5A and induces DAP12 phosphorylation in macrophages. In addition, JEV activates macrophages to secrete proinflammatory cytokines and chemokines, which are dramatically reduced in JEV-infected *Clec5a^−/−^* macrophages. Although blockade of CLEC5A cannot inhibit JEV infection of neurons and astrocytes, anti-CLEC5A mAb inhibits JEV-induced proinflammatory cytokine release from microglia and prevents bystander damage to neuronal cells. Moreover, JEV causes blood-brain barrier (BBB) disintegrity and lethality in STAT1-deficient (*Stat1* ^−/−^) mice, whereas peripheral administration of anti-CLEC5A mAb reduces infiltration of virus-harboring leukocytes into the central nervous system (CNS), restores BBB integrity, attenuates neuroinflammation, and protects mice from JEV-induced lethality. Moreover, all surviving mice develop protective humoral and cellular immunity against JEV infection. These observations demonstrate the critical role of CLEC5A in the pathogenesis of Japanese encephalitis, and identify CLEC5A as a target for the development of new treatments to reduce virus-induced brain damage.

## Author Summary

Japanese encephalitis (JE) is one of the most common forms of viral encephalitis worldwide, and the common complication post viral encephalitis is permanent neuropsychiatric sequelae resulting from severe neuroinflammation. However, specific treatment to inhibit JEV-induced neuroinflammation is not available. We found that JEV interacts directly with CLEC5A, a C-type lectin expressed on the myeloid cell surface. This observation led to two major findings; first, we demonstrate that JEV activates macrophages and microglia via CLEC5A, and blockade of CLEC5A reduces bystander neuronal damage and JEV-induced proinflammatory cytokine secretion from macrophages and microglia. Second, peripheral administration of anti-CLEC5A mAb does not only inhibit JEV-induced BBB permeability, but also reduces the numbers of activated microglia and cell infiltration into the CNS. The attenuation of neuronal damage and reduced viral load correlate with the suppression of inflammatory cytokines TNF-α, IL-6, IL-18, and MCP-1 in the CNS. Our studies provide new insights into the molecular mechanism of neuroinflammation, and reveal a possible strategy to control neuroinflammation during viral encephalitis.

## Introduction

The *Flavivirus* genus includes the mosquito-borne dengue, Japanese encephalitis and yellow fever viruses [1], infections of which can result in clinical syndromes such as hemorrhagic fever and encephalitis. There are four serotypes of dengue virus (DV), which can give rise to severe hemorrhagic syndrome (dengue hemorrhagic fever/DHF) and capillary leakage induced-hypovolemic shock (dengue shock syndrome/DSS) [2]. On the other hand, the Japanese encephalitis virus (JEV) serological group, which includes West Nile virus (WNV) and St. Louis encephalitis virus, is a major contributor to the occurrence of viral encephalitis worldwide [3], with 50,000 new cases and 15,000 deaths per annum [4]. JEV is the most prevalent cause of encephalitis and although both inactivated [5] and live-attenuated [6] JEV vaccines have been used in Asia for decades, these are not completely effective against all the clinical isolates [7], and there are still ∼35,000 reported cases of Japanese encephalitis (JE) resulting in 10,000 deaths each year [8]. Unlike DHF and DSS, JE victims experience permanent neuropsychiatric sequelae, including persistent motor defects and severe cognitive and language impairments [9]. However, the molecular pathogenesis of JEV infection is still unclear.

JEV-specific infiltrating T lymphocytes and JEV-neutralizing IgM and IgG are believed to play major roles in the recovery and clearance of the virus, while microglia were shown to secret massive amounts of cytokines following JEV infection [10]. While JEV infects and kills neuron directly [11], viral replication within microglia/glia leads indirect neuronal killing via secretion of cytokines (such as TNF-α) and soluble mediators to cause neuronal death [11]. One of the key factors in indirect neuronal cell death during JE is the uncontrolled overactivation of microglia cells [12]. However, the molecular mechanism of JEV-induced microglia activation is unclear, thus we are interested to identify the key molecule to regulate JEV-induced proinflammatory cytokine release from microglia. This information may help in the development of specific treatments for JEV-induced neuroinflammation.

CLEC5A (also known as myeloid DAP12-associating lectin (MDL-1) [13]) contains a C-type lectin-like fold similar to the natural-killer T-cell C-type lectin domains, and associates with a 12-kDa DNAX-activating protein (DAP12) [14] on myeloid cells such as monocytes, macrophages and neutrophils, but not monocyte-derived dendritic cells. Moreover, we have shown dengue virus (DV) can bind and activate CLEC5A and induce the phosphorylation of DAP12 [15], which is responsible for CLEC5A/MDL-1-mediated signaling [13]. Unlike conventional C-type lectin receptors (CLRs), such as DC-SIGN/CLEC4L, DC-SIGNR/CLEC4M, and mannose receptor/CLEC13D/CD206 [16], which are all involved in dengue virus (DV) entry into target cells, CLEC5A regulates virus-induced proinflammatory cytokine release from macrophages [15]. In addition, blockade of CLEC5A can prevent autoimmune inflammation in collagen-induced arthritis via downregulating osteoclast activation, suppressing cell infiltration of joints, and attenuating proinflammatory cytokine release [17]. These observations indicate that CLEC5A is a critical molecule to regulate inflammatory reactions triggered by pathogens and autoantigens. We thereby went on to determine whether CLEC5A is involved in JEV-induced proinflammatory cytokine release from microglia and bystander neuronal damage. Here, we demonstrate that JEV infects and replicates in peripheral macrophages and microglia. Moreover, blockade of CLEC5A dramatically reduces bystander neuronal damage and JEV-induced proinflammatory cytokine secretion from macrophages and microglia. Furthermore, peripheral administration of anti-CLEC5A mAb attenuates neuronal cell death, inhibits JEV-bearing infiltrating cells into CNS, and restores the expression of tight junction proteins and BBB integrity. These results suggest that CLEC5A is a promising therapeutic target to control neuroinflammation during viral encephalitis.

## Results

### JEV activates macrophages to secrete proinflammatory cytokines via CLEC5A

Reverse-transcription PCR (RT-PCR) using cDNA templates from the human microglial cell line CHME3, macrophage-like cell line U937, and CD14^+^-derived macrophages (MoM) revealed the presence of a dominant transcript (CLEC5A) and an alternatively spliced variant (CLEC5A_S) lacking 23 amino acid (aa) residues within the stalk region of CLEC5A (Figure S1A & S1B). Similarly, murine CLEC5A_S (mCLEC5A_S) lacks 25 aa from the corresponding region of murine CLEC5A (mCLEC5A) [18].

**Figure 1:**
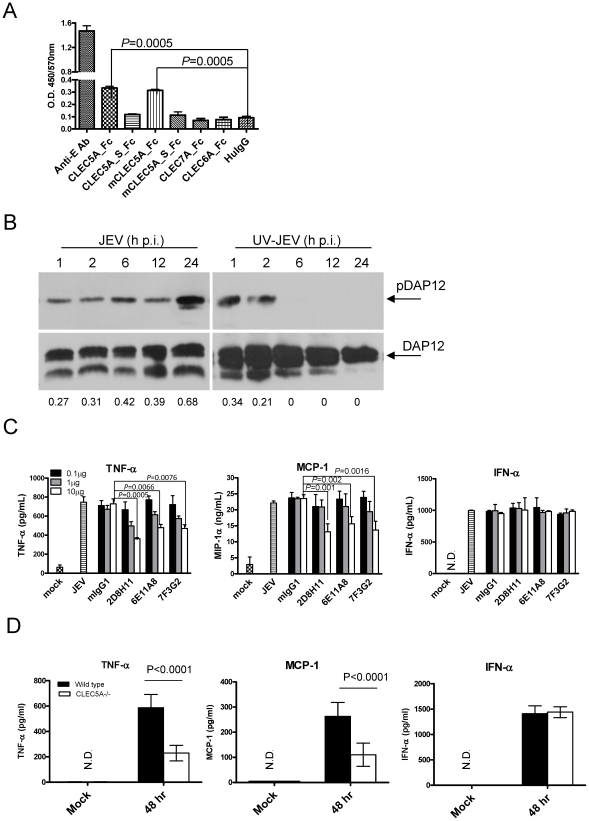
JEV interacts with CLEC5A and induces cytokine secretion via CLEC5A. (**A**) Interaction of JEV with human and murine CLEC5A.Fc fusion proteins was determined by ELISA. Anti-E Ab is a positive control to confirm the capture of JEV particles; HuIgG1: human IgG1. Data are expressed as means ± s.e.m. for three independent experiments; two-tailed Student's t-tests were performed. (**B**) The kinetics of DAP12 phosphorylation induced by JEV (m.o.i. = 2) and ultraviolet-inactivated JEV (UV-JEV) in human macrophages were determined by western blotting (h.p.i., hours post infection). The intensities of pDAP12 bands were quantified with MetaMorph software (Molecular Devices) and its relative intensity to that of the band of respective corresponding DAP12 and are shown under each picture. (**C**) Different amounts of antibody 0.1 µg (2 µg/ml; 0.013 µM), 1 µg (20 µg/ml; 0.13 µM) and 10 µg (200 µg/ml; 1.3 µM) were incubated with MoM before addition of JEV. Dose-dependent inhibition of cytokine release from JEV-infected MoM by anti-CLEC5A mAbs (clones: 2B8H11, 6E11A8, 7F3G2) were determined using ELISAs at 48 h post infection. mIgG1 was used as an isotype control. (D) Bone marrow-derived macrophages (5×10^5^ cells/well) from *Clec5A^−/−^ Stat1^−/−^* and *Clec5A^+/+^ Stat1^−/−^* mice were infected with JEV (m.o.i. = 2) and supernatants were harvested at 48 hr after JEV infection for cytokine measurement. Data were collected and expressed as mean ± s.e.m. from at least three independent experiments. Two-tailed Student's t-tests were performed.

ELISA showed that human CLEC5A and mCLEC5A, but not the alternatively spliced variants or structurally related members of the CLEC family, are able to interact with JEV (Figure 1A). The differential abilities of CLEC5A and CLEC5A_S to bind JEV were further confirmed by immunoprecipitation (Figure S1C). This observation demonstrates that the stalk region of CLEC5A plays a critical role in binding to JEV. Incubation of macrophages with JEV was shown to induce the phosphorylation of DAP12 (Figure S1D), a CLEC5A-associated adaptor protein with an ITAM motif contributing to signal transduction. While UV-inactivated JEV-induced DAP12 phosphorylation lasted for only 2 h, DAP12 phosphorylation was detectable for at least 24 h following infection with live JEV, indicating DAP12 phosphorylation is enhanced by JEV replication (Figure 1B). Compared to live JEV, UV-inactivated JEV only induced transient DAP12 phosphorylation and low amounts (less than 50 pg/ml) of TNF-α and IL-6 secretion (data not shown). This indicates that virus particles released from JEV-infected macrophages can continually activate DAP12 and induce cytokine release. Furthermore, knockdown of CLEC5A using the short hairpin RNA (shRNA) pLL3.7/CLEC5A [15] abolished DAP12 phosphorylation (Figure S1E), suggesting that JEV-triggered DAP12 phosphorylation is mediated via CLEC5A.

We further investigated whether JEV could also infect and activate human CD14^+^-monocyte derived macrophages (MoM). Although JEV was found to be less efficient than DV in infecting macrophages, only JEV infected the human neuroblastoma cell line HTB11 (Figure S2A), suggesting that JEV is both myelotropic and neurotropic. Moreover, JEV activates MoM to secret proinflammatory cytokines, and anti-CLEC5A mAb blocked the release of TNF-α and MCP-1 but not IFN-α, from JEV-infected MoM (Figure 1C) and murine bone marrow-derived macrophages (BMM) from *Stat1* ^−/−^ mice (Figure S2B&S2C). The critical role of CLEC5A in JEV infection was further confirmed by incubating JEV with BMM derived from *Stat1* ^−/−^/*Clec5a* ^−/−^ mice (Figure S3). Compared to *Stat1* ^−/−^/*Clec5a* ^+/+^ mice, BMM from *Stat1* ^−/−^/*Clec5a* ^−/−^ mice secreted significantly less TNF-α and MCP-1 in response to JEV infection, while levels of IFN-α secretion were very similar (Figure 1D). These findings further confirm the critical role of CLEC5A in the JEV-induced inflammatory reaction.

### Blockade of CLEC5A inhibits JEV-induced neuronal death in mixed glial cell culture

**Figure 2:**
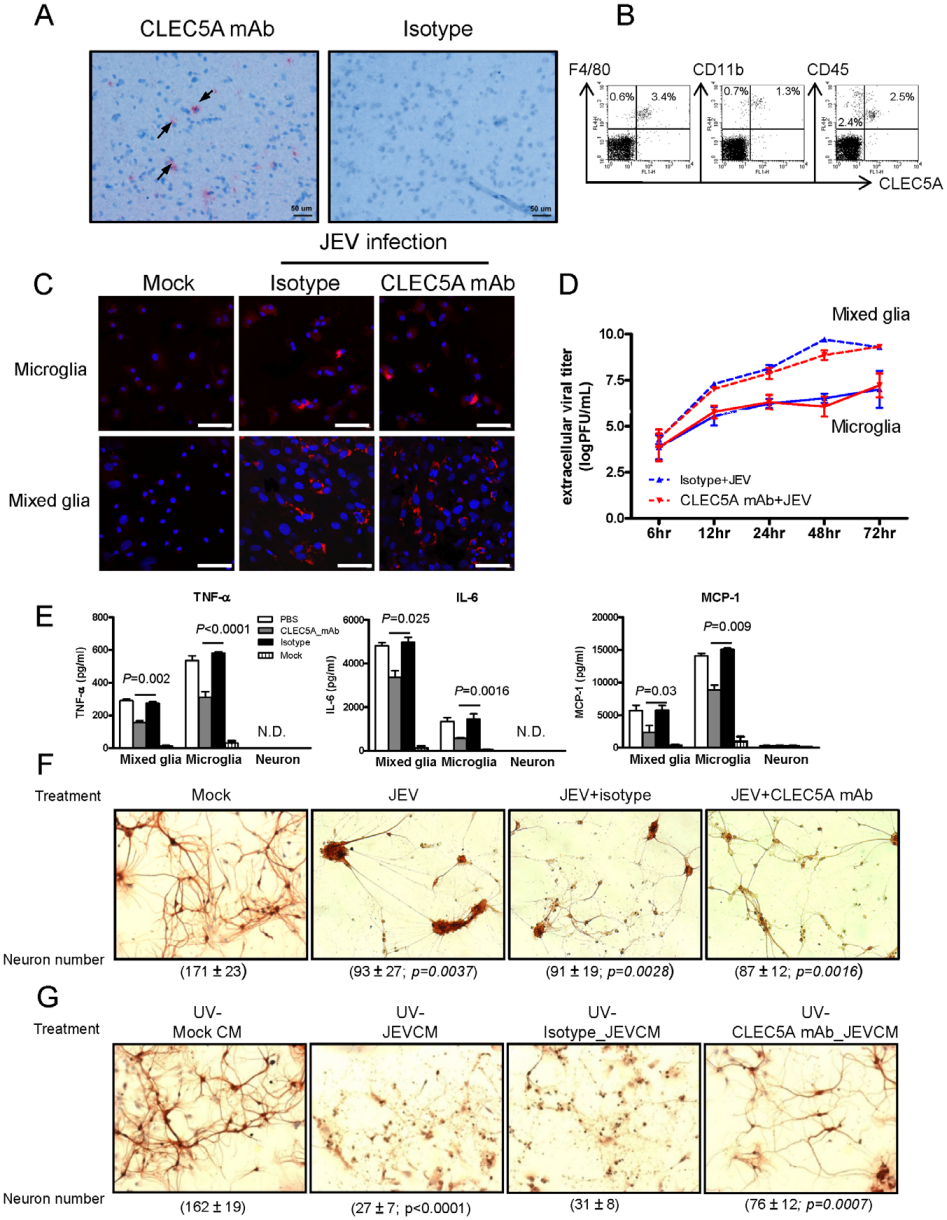
Blockade of CLEC5A inhibits neuronal death induced by supernatant from JEV-infected *Stat1^−/−^* murine mixed glial cell culture. (**A**) Tissue sections of human perilesional brain cortex from glioblastoma multiforme were stained with CLEC5A mAb (20 µg/ml) and isotype control. CLEC5A staining was observed under a light microscope (Nikon) and photographed. (**B**) Detection of CLEC5A^+^ cells in F4/80^+^, CD11b^+^ and CD45^+^ mononuclear cells isolated from naïve murine brain. (**C**) Viral antigen NS3 (red; cells counterstained with Hoechst (blue)) was detected in JEV-infected microglia or mixed glia (m.o.i. = 5) in the presence of anti-CLEC5A mAb (clone: 3E3G4; 200 µg/ml, 1.3 µM) at 48 h post infection. Scale bars, 50 µm. (**D**) The kinetics of virus replication were unaffected by anti-CLEC5A mAb (3E3G4). Viral titers in culture supernatants were determined by plaque assay. Data were collected from three independent experiments. (**E**) anti-CLEC5A mAb (clone: 3E3G4; 200 µg/ml, 1.3 µM) were incubated with mixed glia or microglia before addition of JEV. Cytokine secretion from JEV-infected mixed glia and microglia was harvested and analyzed by ELISA at 48 h p.i.. (**F**) An immunocytochemical analysis (anti-tubulin β III isoform mAb) shows the morphology of JEV-infected neurons (m.o.i. = 5) in the presence of anti-CLEC5A mAb or isotype matched control at 24 h post JEV infection. (**G**) An immunocytochemical analysis (anti-tubulin β III isoform mAb) shows the morphology of neurons after incubation with UV-inactivated conditioned medium (CM) from JEV-infected mixed glia (UV-JEVCM) (m.o.i. = 5), in the presence of isotype matched control (UV-isotype_JEVCM) or anti-CLEC5A mAb (UV-CLEC5A mAb_JEVCM) for 24 h. The morphology of neurons was observed by immunocytochemical staining with anti-Tubulin β III isoform Ab (TU20), and visualized under a light microscope (Nikon). Five fields of views were randomly photographed, and the numbers of live neurons were counted and represented as mean ± s.e.m. (under each picture) for three independent experiments. Significance compared to mock treatment was tested using a two-tailed Student's t-test (Figure 2F). In Figure 2G, the *P<0.0001* indicates a significant difference in UV-MOCK CM vs. UV-JEVCM; *P = 0.0007* indicates a significant difference in UV-Isotype JEVCM vs. UV-CLEC5A mAb JEVCM.

Since astrocytes (glial cells) and microglia (cerebral residential macrophages) are major sources of proinflammatory cytokines during cerebral inflammation [11], [12], [19], we went on to investigate the role of CLEC5A in JEV-induced neuroinflammation in *Stat1* ^−/−^ microglia and mixed glia cells. We found that CLEC5A is expressed on microglia (Figure 2A) as determined by immunohistochemistry staining. We further isolated the mononuclear cells (including hematopoietic and non-hematopoietic cells) by Percoll-gradient centrifugation [20] from naïve mice to determine cell lineages expressing CLEC5A. We found CLEC5A is expressed on the surface of F4/80^+^ tissue macrophages (90%), CD11b^+^ myeloid cells (65%), and CD45^+^ cells (50%; predominantly in CD45^low^ hematopoeitic) (Figure 2B).

Due to the resistance of wild type mice to JEV infection, previous studies in relation to JEV-induced brain damage have utilized intracranial (i.c.) injection of JEV to deliver virus directly to the CNS [21], [22]. However, this approach causes mechanical damage to the BBB and thus cannot be used to address the mechanisms of JEV-induced changes in BBB permeability and the associated entry of virus into the CNS. Since *Stat1* ^−/−^ mice are sensitive to JEV infection even without i.c. puncture, and are able to develop protective immunity after JEV challenge, these animals provide a useful model to test the protective effects of vaccines against JEV and other pathogens which are unable to infect wild type mice. We set up microglia (95% purity) and mixed glial cell (approximately 85% astrocytes and 10% microglia) cultures from *Stat1* ^−/−^ mice to assess the potential involvement of CLEC5A in regulating cerebral inflammation and neuronal death after JEV infection (Figure S4). Immuno-staining with a mAb to JEV nonstructural protein 3 (NS3) showed that ∼30% of mixed glial cells and ∼10% of microglia were infected with JEV. However, anti-CLEC5A mAb was unable to inhibit either NS3 expression (Figure 2C) or virus replication (Figure 2D), suggesting that CLEC5A was not involved in JEV entry into these cells.

It has been reported that both JEV-infected astrocytes and microglia release multiple bio-active factors, thereby giving rise to secondary glial activation and neuronal injury [11], [12]. We therefore investigated the release of proinflammatory cytokines from JEV-infected neurons, mixed glia and microglia cultures. Cells were preincubated with anti-CLEC5A mAb for 1 h, followed by incubation with JEV at 37°C for 1 h, and supernatants were harvested to determine cytokine release at 24 h post-infection. We found that TNF-α and MCP-1 were produced primarily by microglia, and abundant IL-6 was released by JEV-infected mixed glia (Figure 2E), while JEV-infected neurons only released trace amount of these cytokines (Figure 2E). This observation is in accord with a previous report that JEV-infected astrocytes produce IL-6, but not TNF-α [11]. In our study, pre-incubation with the anti-CLEC5A mAb mediated significant reductions in cytokine release (Figure 2E); we therefore investigated whether anti-CLEC5A mAb could prevent neuronal death during JEV infection. Direct incubation of neurons with JEV caused 40% cell damage (live cell count reduced from 171±23 to 93±27) (Figure 2F), while UV-irradiated conditioned media (in the absence of anti-CLEC5A mAb) from JEV-infected mixed glia (UV-JEVCM; from Figure 2E) caused 80% cell damage (from 162±19 to 27±7 live cells) (Figure 2G). This indicated that soluble mediators released from JEV-infected mixed glia are more toxic than JEV per se to neurons. In addition, anti-CLEC5A mAb protected approximately 50% of neurons from UV-JEVCM-induced cell damage (from 162±19 to 76±12 live cells), but was ineffective in protecting neurons from direct JEV infection (Figure 2F). Previous studies demonstrated that TNF-α released by microglia plays a critical role in JEV-associated neurotoxicity [11], while MCP-1 secreted by microglia can recruit inflammatory cells to cause neuronal damage [12]. Therefore, the anti-CLEC5A mAb-mediated protective effect may occur via blocking the release of TNF-α, MCP-1, and other yet-defined bio-active factors from JEV-infected mixed glial cells and microglia.

### CLEC5A regulates JEV–induced BBB disintegrity

**Figure 3:**
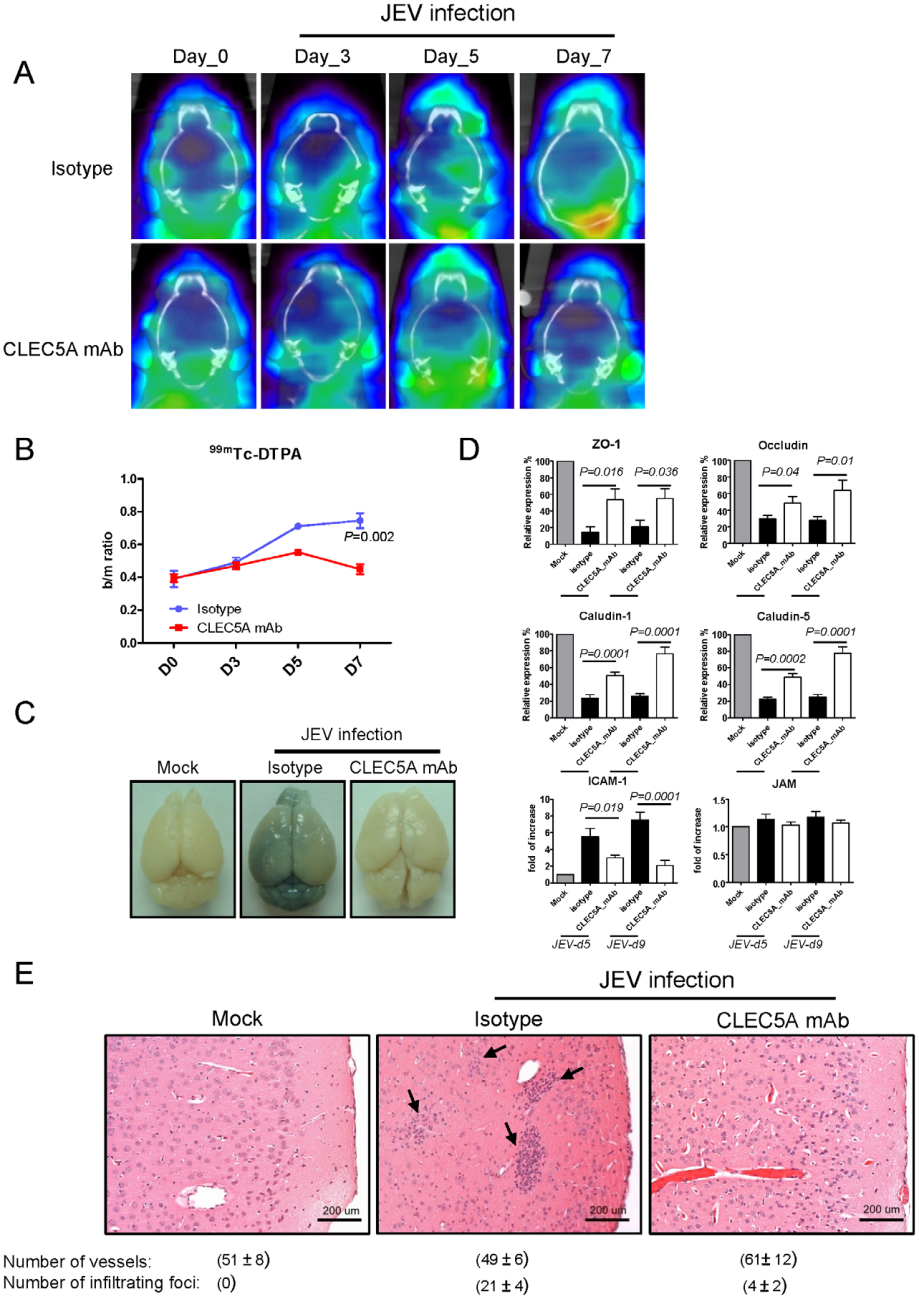
Effect of anti-CLEC5A mAb on dynamic changes in the BBB during JEV infection. (**A**) *Stat1^−/−^* mice were injected intravenously with ^99m^Tc-DTPA to enable brain SPECT/CT imaging, before (D0) or after JEV challenge (100 pfu/mouse), in the presence of anti-CLECA5 mAb (3E3G4) or an isotype matched control. Antibody (150 µg/mouse) was administrated intraperitoneally on days 0, 2, 4, 6 and 8 to determine any protection effects in reduction to BBB permeability. Image datasets were reconstructed using the ordered-subset expectation maximization algorithm with standard-mode parameters. (**B**) The extent of BBB breakdown was calculated as the ratio of the mean counts/pixel in the region of the brain with the greatest accumulation of radiotracer divided by the mean counts/pixel in the neck muscle (b/m ratio). (**C**) Changes in BBB permeability at day 7 post JEV infection (100 pfu/mouse) were determined by Evans Blue assay. (**D**) Brains of JEV-infected mice (n = 5) with isotype control or anti-CLEC5A Ab treatment were harvested at day 5 and day 9 post infection to analyze the expression of transcripts encoding tight junction proteins and adhesion molecules by quantitative real-time PCR. For tight junction proteins, y-axis units represent the expression level of each target gene relative to mock control after internal control normalization; the expression level of adhesion molecules is displayed as fold increase relative to mock control. Two-tailed Student's t-tests were performed. (**E**) H&E staining of murine cerebral cortex at day 5 after JEV infection revealed the inhibitory effect of anti-CLEC5A mAb on perivascular cuffing. Scale bars, 200 µm. Five random fields of views in medium power field (original magnification (OM)×200) were photographed, and the numbers of foci and vessel cross sections in each sample were counted, summed up and represented as mean ± s.e.m. (under each picture) of four independent experiments.

While wild type mice only respond to high dose JEV challenge (1×10^5^ pfu/mouse) with intracranial (i.c.) puncture (Figure S5A), *Stat1* ^−/−^ mice are sensitive to low dose JEV-induced lethality without the need for i.c. puncture (Figure S5B). Thus *Stat1* ^−/−^ mice were used as an *in vivo* model system to test whether anti-CLEC5A mAb could maintain BBB integrity, which is known to be damaged by virus-induced neuroinflammation [23], [24]. Changes in BBB integrity over time following JEV challenge, as revealed by ^99m^Tc-DTPA brain SPECT/CT imaging, showed that JEV infection gave rise to increased BBB permeability from day 3 to day 7 post infection (Figure 3A&B). This effect was reduced (during day 3 to day 5) and normal BBB permeability was restored on day 7 in response to treatment with anti-CLEC5A mAb. The integrity of the BBB after anti-CLEC5A mAb treatment on day 7 was further confirmed by the Evans blue assay (Figure 3C). Since tight junctions are critical in the regulation of BBB permeability, and their disruption is a hallmark of CNS abnormalities [25], we measured the expression of tight junction proteins (such as ZO-1, Occludin and Claudins) and of adhesion molecules (such as JAM-1 and ICAM-1) known to be important for recruitment of inflammatory cells. In JEV-infected mice (treated with isotype mAb), the expression of ZO-1, Occludin, Claudin-1, and Claudin-5 was downregulated, while the expression of ICAM-1 was upregulated. However, the expression of junctional adhesion molecule (JAM-1) was not changed after JEV infection (Figure 3D). Interestingly, anti-CLEC5A mAb restored the expression of tight junction proteins and suppressed JEV-induced upregulation ICAM-1 (Figure 3D). Moreover, the increased BBB permeability in JEV-infected mice was accompanied by perivascular cuffing, while anti-CLEC5A mAb reduced the numbers of infiltrating foci (Figure 3E). This demonstrates that peripheral administration of anti-CLEC5A mAb is able to restore BBB integrity to prevent cell infiltration.

### Blockade of CLEC5A attenuates myeloid cell infiltration into CNS

**Figure 4:**
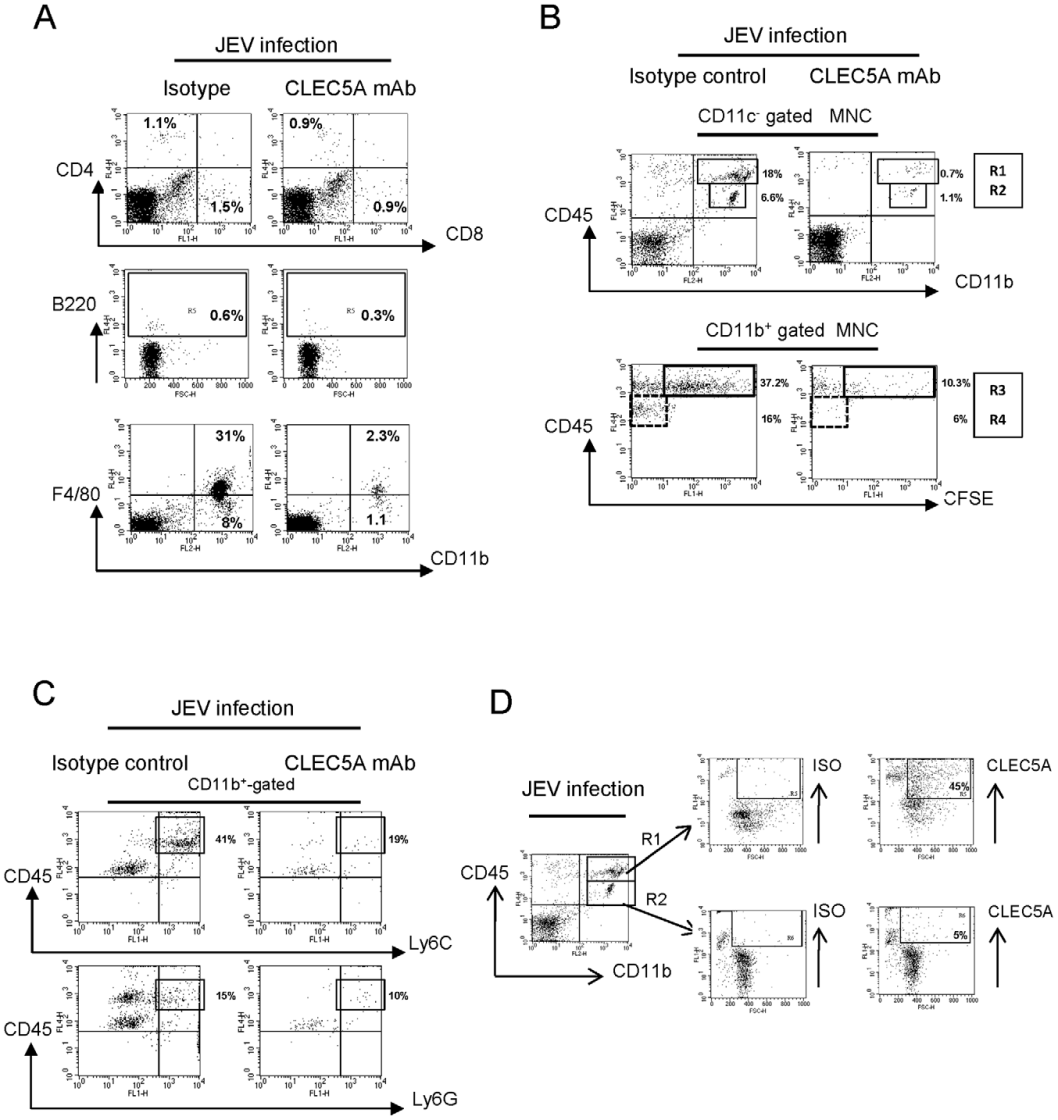
Effect of anti-CLEC5A mAb on recruitment of immune cells into the CNS during JEV-infection. (**A**) Flow cytometry analysis of mononuclear cells (MNCs) isolated from JEV-infected murine brain tissue with isotype control or anti-CLEC5A mAb treatment at day 5 post infection; cell populations were determined by staining specific cell surface markers. (**B**) MNCs were further characterized by determining expression levels of CD45 in the CD11b^+^CD11c^−^ population to distinguish inflammatory myeloid cells from peripheral (R1, CD45^hi^) and resident microglia (R2, CD45^low^), respectively (upper panel). Recruitment of CD11b^+^CD45^+^ cells into brain of JEV-infected mice was tracked by in vivo labeling of blood cells with fluorescent dye CFSE. (**C**) Analysis of the CD45^+^CD11b^+^ population for Ly6C and Ly6G expression revealed the major Ly6C^+^ and minor Ly6G^+^ populations in CD11b^+^CD45^hi^ peripheral inflammatory myeloid cells. The percentages indicate the specific region in CD11b^+^-gated cells (the collected CD11b^+^ cells in the isotype group and CLEC5A mAb treatment group are approximately 25,000 cells and 1,000 cells, respectively.) Three independent experiments with three mice per group were performed and representative FACS plots are shown. (**D**) CLEC5A expression in peripheral inflammatory myeloid cells (R1-gated; CD11c^−^CD11b^+^CD45^hi^) and residential microglia (R2-gated; CD11c^−^CD11b^+^CD45^low^) isolated from JEV-infected murine brain.

We further isolated mononuclear cells (MNCs) from the JEV-infected *Stat1* ^−/−^ mice brain by Percoll gradient [20] to analyze the cell lineages. At day 5 after JEV infection, most of the infiltrating cells are F4/80^+^CD11b^+^ myeloid cells (left column, Figure 4A). Peripheral administration of anti-CLEC5A mAb efficiently inhibits the infiltration of F4/80^+^ CD11b^+^ myeloid cells (right column, Figure 4A).

It has been shown that CD11b^+^/CD45^+^ cells contribute to the pathogenesis of West Nile virus (WNV) encephalitis [26], thus triple-color staining (CD11b/CD11c/CD45) was used to gate the CD11c^−^ population to distinguish residential microglia (R1) and infiltrating myeloid cells (R1) (Figure 4B). To determine the source of CD45^+^CD11b^+^ myeloid cells, carboxyfluorescein succinimidyl ester (CFSE) was injected into peritoneum (i.p.) at day 2 post JEV infection to trace the migration of CFSE^+^ cells (Figure S6A). All the peripheral blood cells were labeled with CFSE at day 5 post infection whether treated with isotype or anti-CLEC5A mAb (Figure S6B). In addition, intraperitoneal injection of CFSE was unable to label CNS MNCs without intracranial (i.c.) puncture (mode III, Figure S7B), nor when i.c. puncture was performed at 2 days after i.p. injection of CFSE (mode II, Figure S7B), even though simultaneous CFSE i.p. injection and i.c. puncture (mode I, Figure S7B) can label intracranial MNCs weakly. This indicates that CFSE is either degraded or excreted within 48 hours.

We then analyzed the intracranial MNCs isolated from JEV-infected *Stat1* ^−/−^ mice (Figure 4B). We found that approximately 5×10^4^ and 1.5×10^3^ CD45^+^ cells were found in Percoll-gradient purified MNCs from mice treated with isotype and anti-CLEC5A mAb, respectively. Moreover, anti-CLEC5A mAb was able to suppress the infiltration of CD11b^+^CD45^hi^ cells (R1) into CNS, as well as the proliferation of CD11b^+^CD45^low^ cells (R2) after JEV infection (upper row, Figure 4B). It is interesting to note that all the CD11b^+^CD45^low^ cells are CFSE negative, while most of the CD11b^+^CD45^hi^ cells are CFSE positive (lower row, Figure 4B). These results suggested that the CD11b^+^CD11c^−^CD45^hi^ population (R1) are the infiltrating inflammatory myeloid cells from peripheral blood, while the CD11b^+^CD11c^−^CD45^low^ (R2) are the resident microglia.

It has been demonstrated that CD45^+^CD11b^+^Ly6C^hi^ cells have properties of inflammatory monocytes [27], and CLEC5A^+^CD11b^+^Gr1^+^ cells are responsible for DV-induced septic shock in ConA-primed mice [28], thus we further characterized CD45^+^ populations by detecting the expression of CLEC5A and Gr1 (comprising Ly6C and Ly6G). We found that all the CD45^hi^CD11b^+^ population expresses Ly6C, while 15% of the CD45^hi^CD11b^+^ population also expresses Ly6G (Figure 4C). Moreover, CLEC5A is highly expressed in CD45^hi^CD11b^+^ infiltrating inflammatory myeloid cells (45%), while only 5% of CD45^low^CD11b^+^ cells express CLEC5A (Figure 4D). This observation suggests that the majority of the infiltrating inflammatory cells are CD45^hi^CD11b^+^Ly6C^+^CLEC5A^+^, while peripheral administration of anti-CLEC5A mAb efficiently reduces the numbers of inflammatory myeloid cells and suppresses the proliferation of resident macrophages in the CNS (R2).

### Blockade of CLEC5A attenuates CNS inflammation

**Figure 5:**
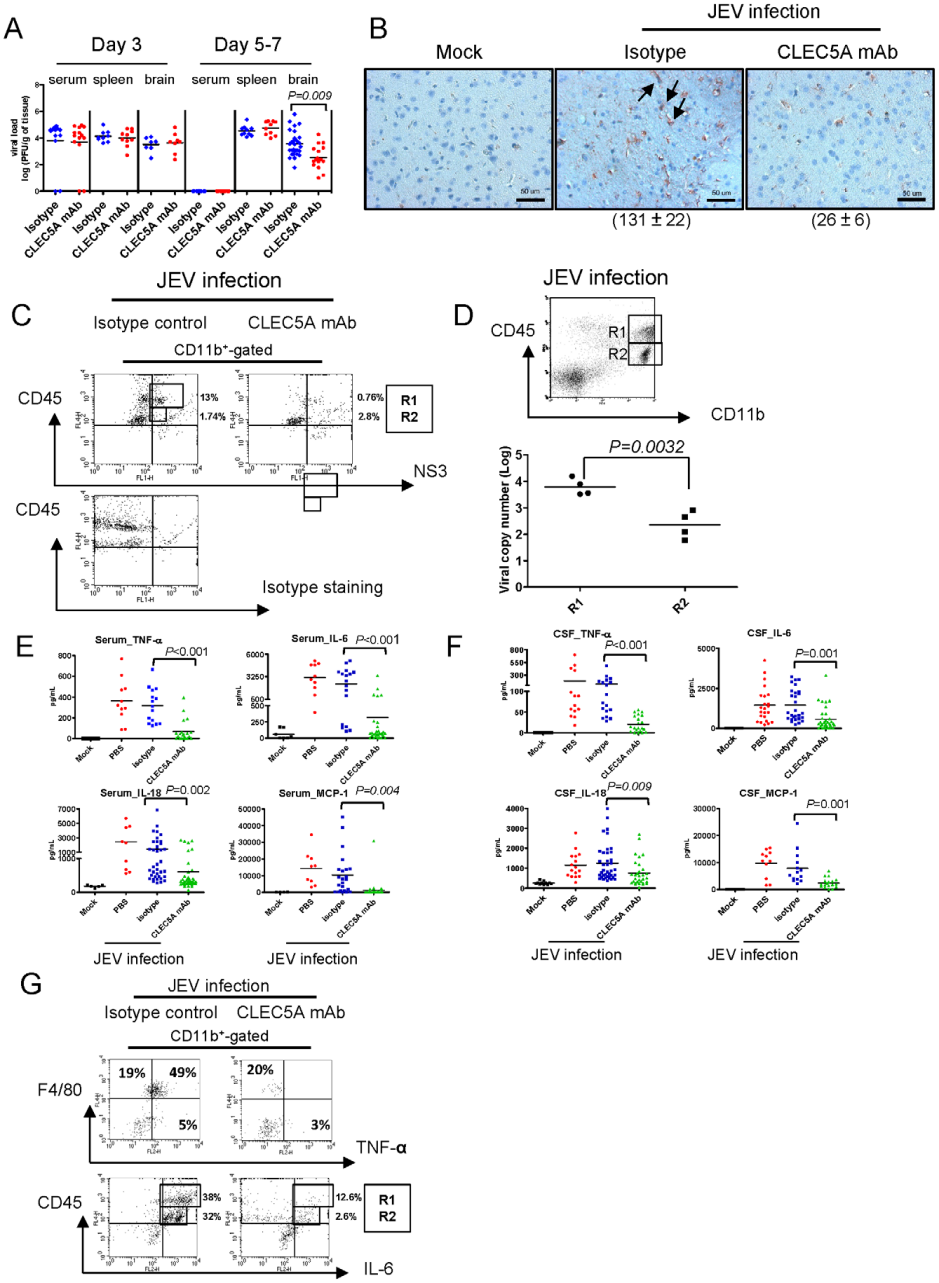
Anti-CLEC5A mAb reduces viral load in the CNS and attenuates CNS inflammation. (**A**) Viral titer was determined by plaque assay for sera and tissue homogenates from JEV infected mice with or without anti-CLEC5A mAb treatment (two-tailed Student's t-tests). (**B**) The extent of NS3 expression in the cortex was diminished after peripheral administration of anti-CLEC5A mAb at day 5 post JEV infection. Scale bars, 50 µm. Five random fields of views in medium power field (original magnification (OM)×200) were photographed, and the numbers of NS3^+^ cells in each sample were counted, summed up and represented as mean ± s.e.m. (under each picture) of four independent experiments. (**C**) Numbers of NS3^+^ leukocytes (CD45^hi^CD11b^+^ and CD45^mid^CD11b^+^) in JEV-infected brain were significantly reduced by administration of anti-CLEC5A mAb at day 5 post JEV infection. (**D**) Quantification of viral copies in CD45^hi^CD11b^+^ (R1) and CD45^low^CD11b^+^ (R2) cells sorted from the brain of JEV-infected mice. The average of total cell numbers in R1 and R2 were approximately 5×10^4^ and 2×10^4^, respectively, in the isotype treated group. Q-PCR assay was performed on total RNA from each population and normalized with its respective internal control. Data were collected from four independent experiments. Two-tailed Student's t-tests were performed. Cytokines levels in the sera (**E**) and CSF (**F**) were measured by ELISA at day 5–7 post JEV infection. The effects of CLEC5A mAb (3E3G4) and isotype control were compared using two-tailed Student's t-tests. (**G**) Intracellular TNF-α and IL-6 were detected in CD11b^+^ gated F4/80^+^ and CD45^+^ cells isolated from JEV-infected brain by flow cytometry.

To determine whether anti-CLEC5A mAb can suppress brain inflammation, viral load was measured in the sera and tissue extracts collected from mice at day 3 and day 5–7 post JEV infection. Short term viremia was observed at day 3 post infection, with all the viruses being cleared from peripheral blood at day 5–7 post infection (Figure 5A). In contrast, JEV titers in spleen and brain were maintained at a high level from day 3 to day 7 post infection. Anti-CLEC5A mAb reduced viral load in the brain at day 5–7 (p = 0.009) but was ineffective in spleen (Figure 5A), correlating with reduced NS3 expression in the brain at day 5 post infection (Figure 5B). While JEV infection increased the numbers of MNCs in brain (from 0.8–1×10^4^ to 1–2×10^5^/brain), anti-CLEC5A mAb reduced the numbers of MNCs (2–3×10^4^/brain) and NS3-bearing infiltrating myeloid cells (R1) and microglia (R2) (Figure 5C). To further confirm the replication of JEV in R1 and R2 populations, reverse-transcription PCR was used to quantitate the JEV copy numbers in each population after sorting by FACS (Figure 5D). As shown in Figure 5D, inflammatory myeloid cells (R1) bear higher copies of viral RNA than resident microglia (R2). This result suggests that anti-CLEC5A mAb is able to reduce neuroinflammation by suppressing the infiltration of JEV-positive myeloid inflammatory cells into CNS.

We also found that JEV induced the release of TNF-α, IL-6, MCP-1 and IL-18 into the peripheral blood and CSF at day 5–7 post infection, whereas anti-CLEC5A mAb caused significant suppression of cytokine levels (Figure 5E&F). Since TNF-α and IL-18 are responsible for the cytotoxic and inflammatory responses in a variety of neuropathological conditions [12], [19], [29], [30], and MCP-1 is a potent chemoattractant for monocytes and dendritic cells [31], reduced cytokine levels in the CNS following anti-CLEC5A mAb treatment would be expected to limit neuronal damage. We found that the populations of TNF-α^+^ (Figure 5G, upper panel) and IL-6^+^ (data not shown) CD11b^+^/F4/80^+^ cells (infiltrated monocytes and resident microglia), as well as IL-6^+^ (Figure 5G, lower panel) and TNF-α^+^ (data not shown) CD11b^+^/CD45^+^ (R1 and R2) cells were also reduced in JEV-infected mice following anti-CLEC5A mAb treatment at day 5–7 post infection. Thus, even though peripheral administration of anti-CLEC5A mAb is unable to inhibit viral replication, it can reduce viral load and attenuate inflammation in the CNS via inhibition of cellular infiltration and proinflammatory cytokine secretion.

### Anti-CLEC5A mAb reduces neuron damage and reduces astrocytosis

**Figure 6:**
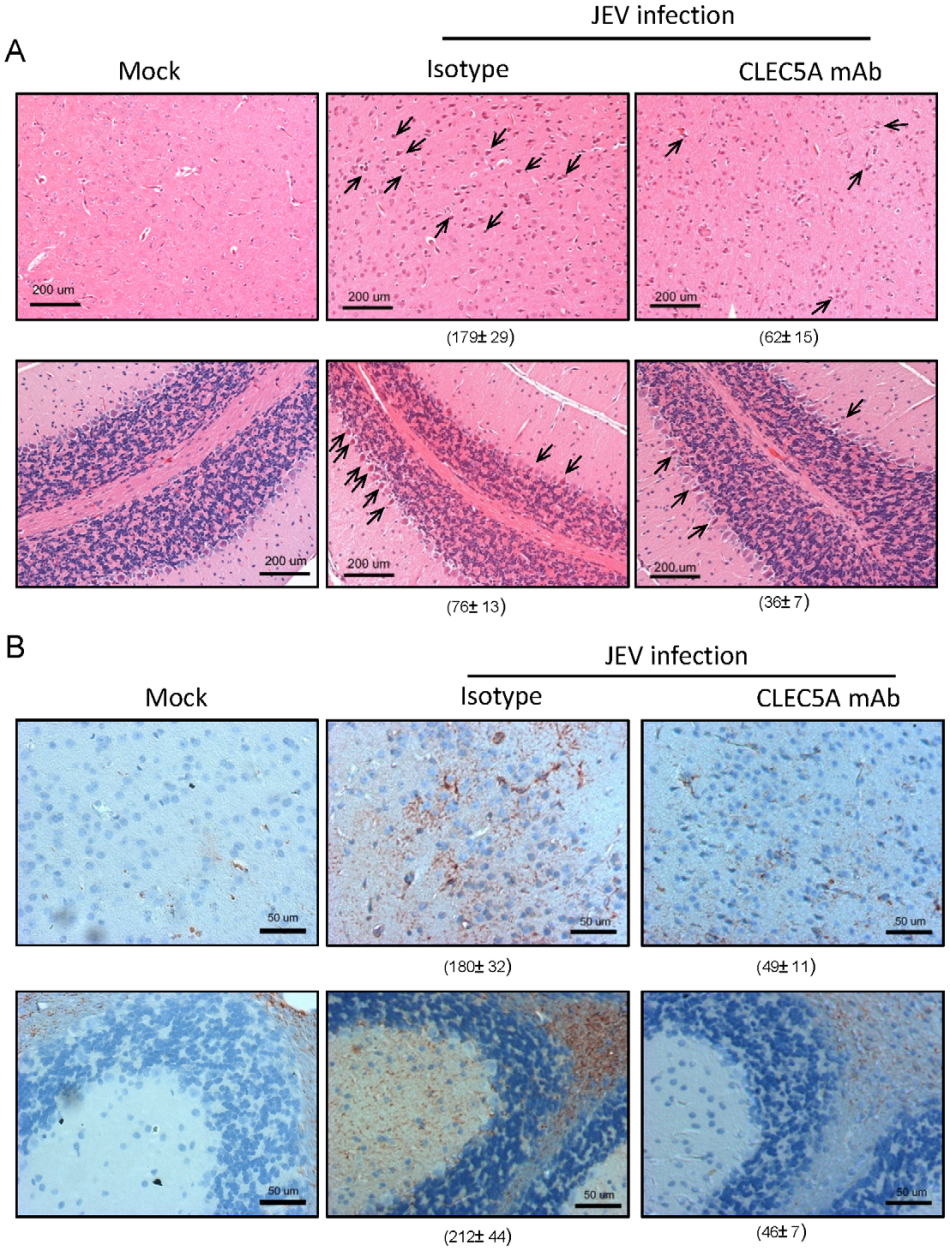
Anti-CLEC5A mAb rescues JEV-induced neuronal damage. (**A**): JEV-induced pathological changes were examined by H&E staining. Arrowheads indicate dysmrophic neurons in the cerebral cortex (upper panel) and damaged Purkinje cells in the cerebellum (lower panel), scale bars, 200 µm. (**B**) Neuronal damage induced-astrogliosis was characterized by GFAP staining, scale bars, 50 µm. For (**A**) and (**B**), tissues were harvested at day 5 post JEV infection. Five random fields of views in medium power field (original magnification (OM)×200) were photographed, and the numbers of dysmorphic neurons or GFAP^+^ cells in cerebral cortex and cerebellum in each sample were counted, summed up and represented as mean ± s.e.m. (under each picture) of four independent experiments.

At day 5 post JEV infection, ischemic, shrunken and damaged neurons and Purkinje cells were observed in the cortical region of the cerebrum (Figure 6A, middle upper panel) and cerebellum (Figure 6B, middle lower panel), respectively. The JEV-induced pathological changes were inhibited by anti-CLEC5A mAb (Figure 6A, right upper & lower panels). Moreover, JEV infection caused astrocytosis, an abnormal increase in the number of astrocytes due to the destruction of nearby neurons in the CNS, in cerebrum and cerebellum as determined by glial fibrillary acidic protein (GFAP) staining (Figure 6B). Upregulation of GFAP and increased astrocyte proliferation in cerebrum (Figure 6B; middle upper panel) and cerebellum (Figure 6B; middle lower panel) were observed in mice treated with an isotype-matched control antibody, while anti-CLEC5A mAb downregulated reactive astrocytosis substantially (Figure 6B, right upper & lower panels). Thus, peripheral administration of anti-CLEC5A not only suppresses neuroinflammation, but also increases the survival of neuronal and Purkinje cells.

### Blockade of CLEC5A prevents JEV-induced lethality and allows development of adaptive immunity against JEV

**Figure 7:**
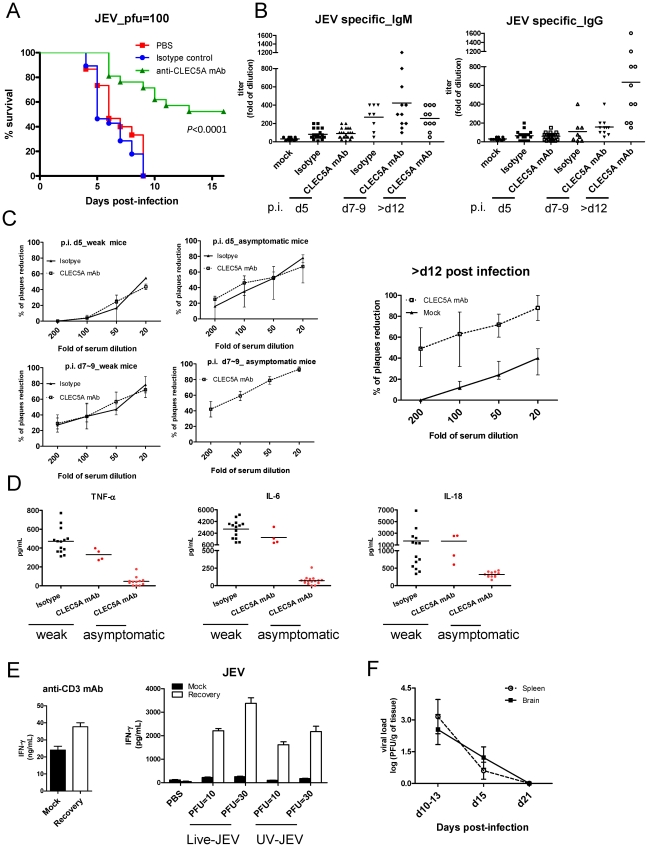
Anti-CLEC5A mAb protects mice from JEV-induced lethality. (**A**) Survival of *Stat1* ^−/−^ mice (8–10 weeks) was monitored for 16 days after intraperitoneal inoculation of JEV (100 pfu/mice); data were collected from four independent experiments and are shown as Kaplan–Meier survival curves with log rank test; n = 20 for each group. (**B**) Titers of anti-JEV specific IgM and IgG antibodies in murine sera were determined by ELISA (**C**) Pooled sera from surviving mice were serially diluted and analyzed using a for the plaque reduction neutralization test (PRNT); data were collected from four independents experiments with n = 5 for each group. (**D**) INF-γ secretion from total splenocytes of surviving mice (recovery, day 21 after JEV infection) after incubation with JEV and UV-JEV for 72 h, but not from mock infected mice. (**E**) The ability of IFN-γ-secreting T-cell was verified by immobilized anti-CD3 mAb to activate T cells in both mock and JEV-infected (recovery) mice. (**F**) Viral titers in spleen and brain isolated from surviving mice after JEV challenge were determined by plaque assay.

Investigation of the ability of anti-CLEC5A mAb to protect *Stat1* ^−/−^ mice from JEV-induced lethality revealed that 50% of *Stat1* ^−/−^ mice that succumbed to JEV infection died in the early stages (6 days post infection), and all the mice died within 9 days post infection. In contrast, administration of anti-CLEC5A mAb from day 0 (150 µg/mouse on days 0, 2, 4, 6, and 8) protected mice from early lethality (80% survival), and ∼50% mice survived for at least 16 days post infection (Figure 7A). We went on to determine whether administration of anti-CLEC5A mAb inhibited adaptive immunity against JEV. All the mice that survived JEV-induced lethality were found to have sero-conversion from day 12 post infection (Figure 7B), suggesting that suppression of JEV-induced inflammation by anti-CLEC5A mAb did not prevent development of humoral immunity against the virus. This was further confirmed by plaque reduction neutralization tests (PRNT), where serum from surviving mice efficiently neutralized JEV infection (Figure 7C). In addition, the proinflammatory cytokine levels at day 7–9 post infection were much lower in the sera of asymptomatic mice (Figure 7D). These mice also exhibited JEV-specific T-cell responses; high levels of IFN-**γ** were released from JEV-challenged splenocytes, while IFN-**γ** was almost undetectable in cells from uninfected mice under the same conditions (Figure 7E). It is interesting to note that virus was cleared from the spleen and CNS at day 21 post infection in surviving mice (Figure 7F), suggesting that the host can eradicate JEV after anti-CLEC5A mAb treatment. Thus, blockade of CLEC5A can suppress proinflammatory reactions without interfering with the development of anti-JEV immunity, making CLEC5A a promising target for the treatment of flaviviral infections in the future.

## Discussion

There is growing evidence that microglial cell activation contributes to neuronal damage and neurodegenerative diseases [32]. Following pathogen invasion, these cells play a role in the clearance of cell debris from damaged tissue, but also secret inflammatory cytokines, which are key mediators of the neurodegeneration associated with both acute and chronic CNS pathologies [33]. Our observation that blockade of CLEC5A on microglia attenuates the neuronal damage caused by the supernatants from mixed glia culture in vitro further demonstrates the contribution of microglia to JEV-induced neuronal death. Moreover, peripheral administration of anti-CLEC5A mAb was found to preserve BBB integrity, inhibit cellular infiltration into the brain, and reduce neuronal death *in vivo*. This may be attributed the fact that the anti-CLEC5A mAb can enter the CNS when the permeability of BBB is increased during the acute stage of infection, where intracranial anti-CLEC5A mAb can inhibit microglia activation and attenuate neuroinflammation.

Although JEV can directly infect neurons and cause cell damage, the soluble mediators from CLEC5A^+^-activated microglia and infiltrating myeloid cells seem to be the major cause of neuron damage (bystander neuronal damage), since the inhibition of JEV-induced neuroinflammation by anti-CLEC5A mAb does not arise from interactions with cells, such as astrocytes and gangliocytes in the CNS, or from prevention of neuron damage caused by direct JEV infection. Even though anti-CLEC5A mAb has relatively mild inhibitory effects on cytokine release in JEV-infected macrophages (Figure 1) and microglia (Figure 2), it can inhibit the permeability change of BBB (Figure 3), reduce cell infiltration into the CNS (Figure 3 & 4) and protect mice from JEV-induced lethality. This may due to the blocking effect of anti-CLEC5A mAb to inhibit the release of yet-discovered soluble mediators which are critical to control BBB permeability and neuronal death. The alternative is that anti-CLEC5A mAb can inhibit the infiltration of CLEC5A^+^ MNCs which are pathogenic to CNS, thus suppressing neuronal inflammation and reducing lethality. The possibility of a direct effect on MNCs is supported by the observation that DV can activate CLEC5A^+^ CD11b^+^Gr-1^+^ immature myeloid cells to induce shock in ConA-treated mice [28]. Finally, anti-CLEC5A mAb may block the interaction of CLEC5A^+^ cells with endogenous ligands to reduce neuroinflammation. It has been shown that CLEC5A is critical for collagen-induced autoimmune arthritis (CIA), and CLEC5A-deficient mice are resistant to CIA. This observation suggests that the yet-characterized CLEC5A endogenous ligand(s) is (are) able to activate CLEC5A^+^ cells to induce inflammatory reactions [17]. Therefore, the peripheral administration of anti-CLEC5A mAb may inhibit JEV-induced inflammation via blocking multiple pathways.

It is interesting to note that anti-CLEC5A mAb is able to inhibit the secretion of IL-18, which is one of the key factors responsible for neuroinflammation and neurodegeneration [29]. IL-18 enhances caspase-1 expression and induces the production of matrix metalloproteinase (MMPs) and other proinflammatory cytokines such as TNF-α and IL-1β [34], [35]. Upregulation of IL-18 was detected in the sera and CNS of JEV-infected mice, thus the potent protective effects of anti-CLEC5A mAb in JEV-induced neuroinflammation can be attributed, at least in part, to inhibition of IL-18 secretion in the periphery and in the CNS; i.e., CLEC5A is a critical to regulator of neuroinflammation during JEV infection.

After anti-CLEC5A mAb treatment, approximately 50% of JEV-infected *Stat1* ^−/−^ mice became asymptomatic and survived for at least 21 days post infection, while the others remained weak and died gradually within 12 days. While the titers of the neutralizing antibody were similar in the weak and asymptomatic groups (Figure 7C), the proinflammatory cytokine levels at 7–9 days post infection were much lower in the sera of asymptomatic mice (Figure 7D). This indicates that JEV-induced neuroinflammation is the key parameter in predicting the outcome of JEV infection [19], and that effective suppression of neuroinflammation in the acute stage is critical to increasing survival and preventing neurological sequelae. This observation also suggests that administration of anti-JEV antiserum to patients during the acute stage of infection may be futile, and this argument is supported by the observation that injection of a neutralizing cross-reactive mAb against JEV E protein causes early death in JEV-infected mice [36]. Even though the direct relevance of this *Stat1* ^−/−^ mouse model in relation to human infection with JEV needs to be further confirmed, data for in vivo protection of CLEC5A mAbs still shed light on its therapeutic potential for blocking neuroinflammation.

Previous studies have shown that CLEC5A can interact with all the four serotypes of DV [15], [37], and here we have demonstrated that CLEC5A also interacts with JEV (Figure 1A), but not with other viruses such as influenza virus and EV-71 (Figure S8). In addition, anti-CLEC5A mAb can inhibit inflammatory reactions in human macrophages in response to the structurally related West Nile virus (WNV) (Figure S9). Thus, CLEC5A may play a critical role in the pathogenesis of all the flaviviral infections.

This study has clearly demonstrated that infiltration of inflammatory cells into CNS correlates with increased BBB permeability, suggesting that BBB integrity has a critical role in limiting JEV-induced CNS inflammation. CLEC5A^+^ microglia and infiltrating myeloid cells appear to be essential for JEV-induced inflammation of the CNS and neurological sequelae, and anti-CLEC5A mAb efficiently suppressed the release of proinflammatory cytokines from microglia and macrophages, restored BBB integrity and increased survival after JEV infection in mice. The critical role of CLEC5A in JEV infection was further confirmed by using *Stat1* ^−/−^ *Clec5a* ^−/−^ mice, which are resistant to JEV challenge (Figure S10). Together with our previous observations that anti-CLEC5A mAb is able to prevent DV-induced systemic vascular permeability and protect mice against DV-induced lethal diseases [15]_ENREF_5, the data presented here indicate that CLEC5A plays critical roles in the pathogenesis of flaviviral infections via regulating vascular permeability and suppressing myeloid cell activation.

Like most members of C-type lectin, their exogenous and endogenous ligands are not identified yet. Since crystal structure of CLEC5A reveals that CLEC5A forms homodimer on cell surface [37], thus the binding of JEV to CLEC5A.Fc may similar to JEV to CLEC5A on cell surface, though cells may also expressed other C-type lectin to increase binding affinity. Recently, we have developed an innate immunity receptor-based ELISA (IIR-EIA) to determine the polysaccharide profiling [38] and dengue receptor [15] based on C-type lectin.Fc fusion proteins, thus the identification of CLEC5A as a JEV recognition receptor further suggests that IIR-EIA is an useful tool to identify lectin ligands in the future.

## Materials and Methods

### Ethics statement

Human monocytes were obtained from healthy donors at the Taipei Blood Center of the Taiwan Blood Services Foundation, under a protocol (PM-99-TP-075) approved by the IRB of the Clinical Center of the Department of Health, Taiwan. Written informed consent was obtained from all donors.

All animal studies were performed according to the animal study protocol approved by the Animal Experimental Committee of National Yang-Ming University, and in accordance with the recommendations in the Guide for the Care and Use of Laboratory Animals of the Taiwanese Council of Agriculture. The animal protocol was approved by the Institutional Animal Care and Use Committee (IACUC) of National Yang-Ming University (IACUC #1000519). All surgery was performed under sodium pentobarbital anesthesia, and every effort was made to minimize suffering.

### Reagents

Chemical reagents were purchased from Sigma, culture media/supplements from Invitrogen GIBCO and growth factors from R&D Systems. Anti-CD14 microbeads were from Miltenyi Biotec GmbH, while antibodies for FACS analysis were from BD Pharmingen. Other antibodies were from Upstate Biotechnology (anti-phosphotyrosine, clone 4G10), Santa Cruz (anti-human DAP12 & anti-F4/80), Millipore (anti-tubulin β III isoform (TU-20) mAb), and Cell Signaling (anti-glial fibrillary acidic protein).

### Virus

The neurovirulent (RP-9) strain of Japanese encephalitis virus (JEV), used for both in vitro and in vivo studies, was generated from a Taiwanese isolate, NT109 [39]. *Aedes albopictus* C6/36 cells and the baby hamster kidney fibroblast cell line BHK-21 were used for viral propagation and viral titer determination, respectively. Viral particles were purified from JEV-infected C6/36 cell supernatant by sucrose gradient centrifugation (1/10 volume of the 35% (v/v) sucrose buffer (10 mM Tris-HCL pH 7.5, 100 mM NaCl and 1 mM EDTA), followed by ultra-centrifuging at 32,000 rpm for 3.5 hr, and resuspending the viral pellet in 0.5 ml PBS after removing the supernatant thoroughly.

### CLEC5A-virus interaction

Human and murine pcDNA3.1 CLEC5A.Fc DNA constructs were transfected into 293 FreeStyle cells (Invitrogen), and the recombinant proteins were purified with Protein A beads. To determine the CLEC5A-JEV interaction by ELISA, sucrose-cushion-purified JEV particles (5×10^6^ pfu) were coated on microtiter plates, and bound CLEC5A.Fc fusion proteins (0.05 mg/mL; 100 µL/well) were detected with HRP-conjugated anti-human IgG (Fc) (Jackson Immunoresearch) using 3,39,5,59-tetramethylbenzidine (TMB) (BD Pharmingen) as substrate. For immunoprecipitation, 5 µg of CLEC5A.Fc fusion protein was incubated with purified JEV particles (5×10^6^ pfu) at 4°C for 4 h and then with 10 µ1 of protein A–Sepharose beads for 2 h. The immunocomplex was washed gently before fractionation on SDS–PAGE, followed by transfer onto a nitrocellulose membrane and probing with a mouse mAb specific for JEV envelope protein (E) [39]. Immunoblots were developed with HRP-conjugated anti-mouse IgG (Fab)′2 (ab5887, Abcam), followed by enhanced chemiluminescence detection reagents (Amersham).

### Detection of DAP12 by immunoprecipitation and immunoblotting

Macrophages (1×10^6^) were stimulated with JEV (m.o.i. = 2) for 2 h, followed by resuspension in lysis buffer (50 mM Tris-HCl pH 7.5, 150 mM NaCl, 1% (v/v) Triton X-100, 0.1% (w/v) SDS, 5 mM EDTA, 10 mM NaF, 1 mM sodium orthovanadate, and proteinase inhibitor cocktail (Roche)). Total cell extracts (100 µg) were incubated with rabbit anti-DAP12 polyclonal antibody at 4°C for 4 h and then with Protein A–Sepharose for 2 h. The immunocomplex was washed and fractionated on SDS–PAGE, followed by transfer onto a nitrocellulose membrane and probing with anti-phosphotyrosine antibody. Immunoblots were developed with HRP-conjugated anti-mouse IgG (Cat. AP181P; Chemicon) followed by enhanced chemiluminescence detection reagents ( Immobilon Western; Millipore). To determine the total amount of DAP12 present on the blot, the membrane was stripped with Re-Blot Plus Strong solution (Cat. 2504; Chemicon), and probed with rabbit anti-DAP12 antibody.

### Preparation of macrophages

For human macrophage preparation, peripheral blood mononuclear cells (PBMCs) were isolated from the whole blood of healthy human donors by standard density-gradient centrifugation with Ficoll-Paque (Amersham Biosciences). After centrifugation the buffy coat, containing leukocytes (PBMC) and platelets, was further washed with PBS, and CD14^+^ cells were purified using the VarioMACS technique with anti-CD14 microbeads (Miltenyi Biotec GmbH). Cells were then cultured in complete RPMI 1640 medium supplemented with 10 ng/ml human M-CSF (R&D Systems) for 6 days [15]. For preparation of murine bone marrow-derived macrophages, bone marrow cells were isolated from femurs and tibias and cultured in RPMI 1640 complete medium supplemented with 10% (v/v) fetal calf serum (FCS) and 10 ng /ml of recombinant mouse M-CSF (R&D Systems) for 6–8 days. At day 7, the expression of F4/80 (murine macrophage marker) was examined by fluorescence-activated cell sorting; >90% of cells were F4/80^+^ under these culture conditions.

### Preparation of primary murine neuron and glial cultures

Primary neuron, mixed glia and microglia cultures were prepared from the cerebral cortexes of 1-day-old wild-type or *Stat1* ^−/−^ mice (C57BL/6 background) as previously described [11], [40]. In brief, pooled dissected cortexes were digested in papain solution (1.5 mM CaCl_2_, 0.5 mM EDTA, 0.6 mg/ml papain, 0.05 mg/ml DNase I, and 0.2 mg/ml cysteine in Hanks' balanced salt solution) at 37°C for 20 min to dissociate the cells. After centrifugation at 200×*g* for 5 min, cells were plated on poly-L-lysine coated (20 µg/ml) dishes. For cortical neuron cultures, cells were plated in minimal essential medium supplemented with 10% (v/v) FBS and 10% (v/v) horse serum. One day after seeding, the culture medium was replaced with neurobasal medium supplemented with B27, followed by addition of cytosine arabinoside (10 µM) on the third and fourth days to inhibit non-neuronal cell division *in vitro*. The neuron cultures were used for subsequent experiments after 10–12 days. For mixed glia culture, cells were maintained in DMEM/F12 supplemented with 10% (v/v) FCS; medium was replenished 4 days after plating and changed every 3 days. Cells usually reached confluence within 12–14 days after plating. For microglia cultures, the confluent mixed glial cells were incubated with mild trypsin solution (0.25% (w/v) trypsin, 1 mM EDTA) diluted 1∶4 in DMEM/F12 for 30 min at 37°C to detach the upper layer of astrocytes; 10% (v/v) FCS-containing DMEM/F12 medium was then added to inactivate trypsin and the detached astrocytes were aspirated from the mixed glia cultures. The remaining cells (i.e., microglia) were harvested by incubation with 0.25% (w/v) trypsin solution with vigorous pipetting for 5 min and then were resuspended in DMEM-F12 with 10% (v/v) FCS for at least 2 weeks. Cell identity was determined by immunocytochemical staining using antibodies against tubulin β III isoform, (TU20; Millipore) for neurons, glial fibrillary acidic protein (GFAP; Cell Signaling) for astrocytes, and F4/80 for microglia (Santa Cruz Biotechnology). Mixed glial cultures contained ∼85% astrocytes and ∼10% microglia. Neurons and microglia were >95% pure (Figure S5).

### Infection of cells with virus

Human CD14^+^-monocyte derived macrophages (MoM), murine bone-marrow derived macrophages (BMoM), murine mixed glia and microglial cells were mock-infected or infected with JEV at a multiplicity of infection 5 (m.o.i. = 5). Culture supernatants were harvested to determine virus titer and cytokine secretion by plaque assay and ELISA (R&D Systems), respectively. Conditioned media (CM) from mock or JEV-infected mixed glia were collected and UV-irradiated (254 nm) to inactivate virus. UV-irradiated CM (UV-JEVCM) was mixed with fresh neurobasal medium supplemented with B27, before incubation with neuron cells prior to cytotoxicity assays.

### Generation of antagonistic anti-CLEC5A mAbs

Breeder mice (BALB/c strain) were maintained in the standard animal facility of the National Yang-Ming University. For the production of mAbs, mice were immunized with purified recombinant CLEC5A.Fc fusion protein (five doses of 50 µg per mouse). Lymphocytes from the spleens of immunized mice were fused with mouse myeloma NS-1 cells in the presence of 50% (v/v) polyethylene glycol (PEG1450; Sigma). Fused cells were cultured in HAT selection medium and the medium was refreshed after one week. About 2 weeks after fusion, culture supernatants were screened by ELISA to identify the candidate clones for further analysis by limiting dilution. Anti-CLEC5A mAbs were selected by ELISA-based differential screening, and only those recognizing recombinant CLEC5A.Fc, but not human IgG1, were regarded as positive clones. A similar strategy was used to generate anti-murine (m) CLEC5A mAbs. To select antagonistic mAbs against human CLEC5A and murine CLEC5A, mAbs were incubated with human macrophages and murine bone marrow-derived macrophages (*Stat1* ^−/−^), respectively, in 96-well plates (6×10^4^ cells per well) for 30 min at 37°C, before the addition of JEV (m.o.i. = 5) and incubation at 37°C for 2 h. After washing, cells were incubated for a further 36 h before harvesting the supernatants to measure TNF-α production by ELISA. mAbs capable of inhibiting cytokine release from JEV-infected macrophages were used as antagonistic antibodies for *in vitro* and *in vivo* assays.

### Detection of JEV replication in macrophages and neuronal cells

To detect JEV replication in macrophages, cells were incubated with JEV (m.o.i. = 5) for 1 h, then fixed with 1% (v/v) paraformaldehyde and permeabilized with 0.1% (w/v) saponin, followed by addition of anti-NS3 mAb or isotype-matched control (mIgG1; Sigma) and then FITC-conjugated goat F(ab)′ anti-mouse IgG. Emitted fluorescence was detected by flow cytometry (FACSCalibur platform with CellQuest software (Becton Dickinson)). To determine JEV replication, murine neuronal cells were fixed with 4% (v/v) paraformaldehyde, permeabilized with 0.5% (v/v) Triton X-100 in PBS for 10 min and incubated with blocking buffer (10% (w/v) BSA in PBS) before addition of anti-NS3 mAb (20 µg/ml). After washing, cells were incubated with Cy3-conjugated donkey anti-mouse IgG (Jackson Immuno) and Hochest 33342 to detect NS3 and nuclei, respectively. Cover slips were mounted and observed using an FV-1000 laser scanning microscope (Olympus).

### Immunocytochemical analysis

Cells cultured on coverslips were washed twice with PBS, followed by incubation with 4% (v/v) paraformaldehyde for 2 h, and then permeabilized with 0.5% (v/v) Triton X-100 for 15 min. After washing with PBS, cells were subjected to immunocytochemical staining with HISTOMOUSE-SP KIT (Zymed) according to the supplier's instructions. Briefly, cells were incubated with primary antibodies (including anti-TU20 (1∶300), anti-GFAP (1∶300) or anti-F4/80 (1∶500) antibodies for 2 h at room temperature, followed by incubation with biotinylated secondary antibody and peroxidase-conjugated streptavidin. Cells were then incubated with chromogen, and counterstained with hematoxylin before observation under a light microscope (Nikon).

### H&E and immunohistochemical staining

Mice were transcardially perfused with PBS and brains were removed and placed in 4% (w/v) paraformaldehyde overnight at 4°C, followed by embedding in paraffin wax and processing to generate 5-micron sagittal sections for H&E staining. Before immunohistochemical (IHC) staining, paraffin sections were subjected to antigen retrieval at 100°C for 20 min in 10 mM citric acid (pH 6.0), and endogenous peroxidase activity was quenched with 3% (v/v) H_2_O_2_ for 15 min at room temperature. Immunohistochemical staining was performed using a HISTOMOUSE-SP KIT (AEC) (Zymed) together with anti-GFAP (1∶300) Ab or mouse JEV-NS3 ascites fluid (1∶1000). All images were digitized with Nikon scientific imaging software.

### Administration of antibody to JEV-infected mice

Adult C57BL/6- *Stat1* ^−/−^ mice were used in all experimental procedures. Groups of 8-week-old adult mice were inoculated intraperitoneally with JEV (strain RP-9; 100 pfu per mouse) [22]. Anti-CLEC5A mAb or isotype control (150 µg per mouse) were administrated intraperitoneally (i.p.) on days 0, 2, 4, 6 and 8 after JEV infection, and mice were monitored daily for 16 days to assess morbidity and mortality. All experiments were performed according to the animal study protocol approved by the Animal Experimental Committee of Yang-Ming University.

### Isolation of mononuclear cells from the CNS and flow cytometry analysis

Mononuclear cells (MNCs) were isolated from the brains of mock- or JEV-infected mice as previously described [41]. Briefly, PBS-perfused brains were homogenized in HBSS containing 1% (v/v) FCS and minced with a scalpel before being passed through an 18-gauge needle. Homogenates were suspended in 37% (w/v) Percale (4 ml per brain) and overlaid onto 70% (w/v) Percoll (4 ml) in 15 mL conical tubes; 30% (w/v) Percoll (4 ml) and HBSS (2 ml) were added prior to centrifugation at 200 *g* for 40 min at 20°C. After centrifugation, cells were harvested and washed with PBS to remove residual Percoll. The isolated MNCs were resuspended in FACS buffer, followed by incubation with fluorochrome–conjugated anti-CD4, CD8, B220, CD11b, CD45, F4/80 and CLEC5A mAbs. For intracellular cytokine staining, cells were fixed with 1% (v/v) paraformaldehyde and permeabilized with 0.1% (w/v) saponin, before incubation with specific antibodies. Fluorescence was detected using a FACSCalibur platform with CellQuest software (Becton Dickinson).

### Blood-brain-barrier (BBB) permeability assay

BBB integrity was evaluated using the Evans blue assay [24]. Mice were injected intravenously with 100 µl of 1% (w/v) Evans blue in PBS. One hour later, mice were sacrificed and transcardially perfused with 20 ml of normal saline. Brains were removed and photographed.

### Quantitative real-time PCR analysis

Total RNA was extracted from whole mouse brains using an RNeasy extraction kit (Qiagen) and complementary DNA (cDNA) was synthesized by using RevertAid First Strand cDNA Synthesis Kit (Fermentas, Life Science) according the vendor's suggestions. Quantitative real-time PCR analysis was performed using the LightCycler System SW 3.5.3 (Roche Applied Science) (Fermentas, Life Science), and the level of mRNA expression level was normalized with β-2 microglobulin. Primer sequences for tight junction proteins and adhesion molecule: ZO-1, Occludin, Clauidn-1, Clauidn-5, JAM and ICAM-1 were synthesized as described previously [24], [42]. To quantify the viral copy numbers, a standard curve was generated using pCR3.1/JEV-3′UTR plasmid as template (ranging from 32 pg/L to 32 mg/L; the dilution range is equivalent to the copy number 1×10 to 1×10^7^ copies). JEV specific primer: forward 5′-AAGTTGAAGGACCAACG-3′ (nucleotide 10603–10619); reverse 5′-GCATGTTGTTGTTTCCAC-3′ (nucleotide 10789–10772) [43].

### ^99m^Tc-DTPA brain SPECT/CT imaging

A FLEX Triumph preclinical imaging system (Gamma Medica-Ideas, Inc.) was used for the small-animal SPECT/CT scan acquisition. Each mouse was injected intravenously with 18.5 MBq (0.5 mCi)/0.5 cm^3^ of ^99m^Tc-DTPA and images were acquired 30 min after injection. The mice were scanned first by CT for anatomic coregistration and then by a dynamic SPECT sequence comprising 8 frames. The images were viewed and quantified using AMIDE software (free software provided by SourceForge). A quantitative index of BBB breakdown was defined as the ratio of the mean counts/pixel in the region with disruption of BBB compared with the mean counts/pixel in the neck muscle.

### Plaque reduction neutralization test (PRNT)

To determine the titers of anti-JEV IgM and IgG, serial dilution of JEV-infected mouse sera were added to immobilized JEV particles (4×10^6^ pfu) on microtiter plates, and the bound anti-JEV specific antibodies were detected with HRP-conjugated anti-mouse IgM or anti-mouse IgG secondary antibody using TMB as substrate. The neutralizing activities of mouse sera containing JEV-specific antibodies were determined using a plaque reduction neutralization test. Briefly, 100 pfu of JEV were incubated with serial dilutions of serum samples (20-fold to 200-fold) at 37°C for 1 h, followed by overlaying the virus-serum complex onto BHK21 monolayer. After 1-h adsorption, the virus was removed and BHK21 cells were overlaid with 1% (w/v) agarose in RPMI-1640 and incubated at 37°C for 72 h. Cells were then fixed with 10% (w/v) formaldehyde and stained with crystal violet. The neutralization effect was expressed as the percentage reduction in plaque numbers in the presence of an anti-JEV serum compared to plaque numbers following infection with JEV (100 pfu) alone [39].

### JEV-specific T-cell responses

Total splenocytes from the mock-infected mice and the mice that recovered from JEV infection (day 21 after JEV infection) were isolated and seeded in 96-well (5×10^5^ /well). JEV (PFU = 10 and 30) and UV-inactivated JEV were incubated with total splenocytes for 72 hr, followed by collecting supernatants for IFN-γ measurement. The ability of IFN-γ-secreting T cells was verified by immobilized anti-CD3 mAb to activate T cells.

### Generation of CLEC5A KO mice

Mouse *Clec5a* genomic DNA, 30.7 kb in length (Ensembl Genomics database) containing exons 1–7, was isolated from a 129/Sv genomic DNA BAC library (CITB mouse; Invitrogen, San Diego, CA). In order to generate a targeting vector, the *Clec5a* genomic DNA fragment was inserted into the pL253 plasmid containing a neomycin resistance gene allowing antibiotic selection in ES cells. In the targeting vector, exons 3 to 5 of *Clec5a* were flanked by loxP sequences, which could then be excised upon introduction of a Cre recombinase plasmid into the ES cells. Excision of exons 3–5 was confirmed by Southern blot analysis of ES cell genomic DNA, and this was followed by blastocyst injection to generate *Clec5a* chimeric mice. Genotyping was performed by PCR using the following primers: CU, 5-CCCCAGCATCTTTGTTGTTT-3; FD, 5-CCAGCTAGTGGCTCAGTTCC3; and JD, 5-TTTTCTTCCCCATCCTCTGA-3, which generated a 687-bp WT and a 798-bp knockout (KO) product, respectively. To obtain *Stat1*and *Clec5a* double-KO mice, we crossed *Clec5a* KO mice with *Stat1* KO mice [44]. Interbreeding of *Clec5a^+/−^Stat1^+/−^* mice (F1) generated *Clec5a^+/+^Stat1^−/−^* as well as *Clec5a^−/−^Stat1^−/−^* double KO mice, both of which were used in this study. (Figure S4)

### In situ labeling of peripheral blood cells with CFSE

The in situ labeling method was modified from that previously described [45]. The labeling solution (30 µl of 20 of mM CFSE (Molecular Probes, Eugene, OR)) was mixed thoroughly with 140 µl of 100% ethyl alcohol and 250 µl pyogen-free PBS to avoid precipitation. Typically, 8 µl of labeling was injected per gram of mouse body weight (approximately 3 µg CFSE/g) up to a maximum of 300 µl of solution. To track leukocyte migration, mice were injected intraperitoneally with CFSE solution on day 2 after JEV infection. Blood samples were collected from CFSE-labeled mice to assess the labeling efficiency, and the MNCs in brain were also harvested from perfused mice to analyze and distinguish the infiltrating and resident cells in brain on day 5 after JEV infection. The experimental procedures and labeling control are shown in supplementary Fig. 6 & 7.

### Statistical analysis

Standard errors of the mean (s.e.m.) were calculated and data was analyzed using unpaired Student's *t*-tests. Survival curve comparisons were performed using log-rank tests (Prism software).

## Supporting Information

Figure S1 **JEV interacts with CLEC5A and induces DAP12 phosphorylation via CLEC5A.** (**A**) Expression of human CLEC5A and an alternatively spliced variant (CLEC5A_S) in human microglial cell line (CHME3), monocytic cell line (U937) and CD14^+^-monocyte derived macrophages (MoM) was detected by RT-PCR. (**B**) Schematic representation of human and murine CLEC5A. Sequence analysis of PCR products revealed that human CLEC5A_S lacks 23 amino acids in the stalk region (aa 48–70), while mCLEC5A_S lacks 25 amino acids (aa 28–52) located in stalk regions due to alternative splicing. (**C**) JEV–CLEC5A.Fc complexes were immunoprecipitated with protein A–Sepharose and detected by anti-JEV envelope protein mAb. (**D**) JEV-induced DAP12 phosphorylation (1 h post infection) in human macrophages was determined by western blotting. (**E**) Effects of shRNAs (pLL3.7 backbone) on inhibition of JEV-mediated DAP12 phosphorylation were determined by western blotting (h.p.i., hours post infection). The pLL3.7/CLEC4L was used as a control shRNA.
(TIF)

Figure S2 **JEV replicates in macrophages and induces cytokine release.** (**A**) Human MoM and HTB11, (a human neuroblastoma cell line) infected with DV or JEV (m.o.i. = 5) were subjected to flow cytometry analysis at 48 h post infection using an antibody to nonstructural protein 3 (NS3) to detect viral antigens. (**B**) Murine bone marrow-derived macrophages (BMDM) from wild type and *Stat1* ^−/−^ mice were infected with JEV (m.o.i. = 5), followed by anti JEV-NS3 mAb staining and FACS analysis. (**C**) BMDM from *Stat1* ^−/−^ mice were infected with DV JEV (m.o.i. = 5) in the absence or presence of different doses of anti-CLEC5A mAbs, and supernatants were harvested at 48 h post infection for cytokine determination by ELISA. Data were collected and expressed as mean ± s.e.m. from at least three independent experiments. Two-tailed Student's t-tests were performed.
(TIF)

Figure S3 **Targeting strategy for generation of** ***CLEC5A*** **KO mice and** ***CLEC5A*** **and** ***STAT1*** **double KO mice.** (**A**) Targeting vector for generation of *Clec5a* ^−/−^ mice. A neomycin resistance gene cassette (NEO) was introduced into targeting vector for positive selection; and two loxP sequences (green triangles) flanking exons 3 to 5 allow the removal of CLEC5A exon 3–5 using a Cre-loxP excision system. Locations of PCR primers used for genotyping are shown under targeting vector. (**B**) Genotyping by PCR using CU+FD and CU+JD primer sets for wild type and *Clec5a* ^−/−^ mouse, respectively. (**C**) Double KO mice were produced by mating *Clec5a^+/+^ Stat1^−/−^* and *Clec5a^−/−^ Stat1^+/+^* mice, and the F1 offspring were further interbred to generate F2 offspring. (**D**) Determination of CLEC5A and STAT1 expression in peripheral blood cells by flow cytometry.
(TIF)

Figure S4 **Flow chart for isolation of glia cells and mixed glia fractions from cerebral cortices.** Neurons and mixed glia were prepared from the cerebral cortices of neonatal *STAT1* ^−/−^ mice, and differentiated in neurobasal medium supplemented with B27 (Life Technologies) and DMEM/F12 (Life Technologies) supplemented with 10% (v/v) FCS, respectively. Microglia were further enriched from differentiated mixed glial cell cultures. Neurons, astrocytes and microglia were characterized by staining with antibodies to tubulin β III isoform, glial fibrillary acidic protein (GFAP), or F4/80, respectively. Mixed glial cultures contained ∼85% astrocytes and ∼10% microglia. The purity of neurons and microglia was >95%.
(TIF)

Figure S5 **Murine models for JEV infection.** (**A**) Wild-type C57BL/6 mice (n = 10 per group) were challenged with various doses of JEV (pfu) via an intraperitoneal route with intracranial injection of 30 µL PBS simultaneously (i.p.+i.c.). (**B**) *STAT1*-deficient mice were intraperitoneally infected with a range of doses of JEV (n = 6 per group). All mice were monitored daily for 21 days and outcomes are shown as Kaplan–Meier survival curves with log rank test.
(TIF)

Figure S6 **In situ labeling of peripheral leukocytes with CFSE in JEV-infected mice.** (**A**) Schematic representation of the procedure for in situ labeling with CFSE fluorescence dye in JEV-infected mice. (**B**) Validation of CFSE labeling efficiency by analyzing fluorescence intensity in peripheral blood leukocytes from JEV-infected mice at 72 hr after CFSE injection (day 5 after JEV infection).
(TIF)

Figure S7 **CFSE distribution into CNS after intracranial puncture.** (**A**) Three modes to evaluate the distribution of CFSE after i.p. injection to mice with or without intracranial i.c. puncture. (**B**) Analysis of CFSE fluorescence in the MNCs isolated from brain MNCs using Percoll-gradient centrifugation. MFI of each group was shown in left panel. MFI: mean fluorescence intensity.
(TIF)

Figure S8 **Interactions of CLEC5A with viruses.** Various human viruses (5×10^6^ pfu/well) were immobilized on microtiter plates, followed by incubation of CLEC5A.Fc (0.05 mg/ml; 100 µl/well) and HRP-conjugated goat-anti-human IgG. Specific interactions of CLEC5A with human viruses were determined by addition of TMB as substrate, and measurement of absorbencies at 450 nm.
(TIF)

Figure S9 **WNV replicates in human macrophages and antagonistic anti-CLEC5A mAbs inhibit WNV-induced macrophage activation.** (**A**) Human CD14^+^-monocyte derived macrophages (MoM) infected with WNV (m.o.i. = 5) were subjected to flow cytometry analysis at 48 hr post infection using anti-NS3 antibody to determine WNV replication. (**B**) Dose-dependent inhibition of cytokine release from WNV-infected MoM by anti-CLEC5A mAbs (clones: 3E12A2, 3E1C1 and 9B12H4) determined by ELISA at 48 hr post infection. mIgG1 acts as an isotype matched control. Data were collected from at least three different donors and expressed as mean ± s.d. Two-tailed Student's t-tests were performed.
(TIF)

Figure S10 ***Stat1*** **^−/^** ^***−***^ ***Clec5a*** **^−/−^ mice are resistant to JEV-infection.** Survival of both the *Stat1* ^−/*−*^ *Clec5a* ^+/+^ and *Stat1* ^−/*−*^ *Clec5a* ^−/−^ mice (8–10 weeks) was monitored for 21 days after i.p. inoculation of JEV (100 pfu/mice); data were collected from four independent experiments and are shown as Kaplan–Meier survival curves with log rank test; n = 20 for each group.
(TIF)

## References

[1] Mukhopadhyay S, Kuhn RJ, Rossmann MG (2005). A structural perspective of the flavivirus life cycle.. Nat Rev Microbiol.

[2] Wilder-Smith A, Schwartz E (2005). Dengue in travelers.. N Engl J Med.

[3] Weaver SC, Barrett AD (2004). Transmission cycles, host range, evolution and emergence of arboviral disease.. Nat Rev Microbiol.

[4] Hollidge BS, Gonzalez-Scarano F, Soldan SS (2010). Arboviral encephalitides: transmission, emergence, and pathogenesis.. J Neuroimmune Pharmacol.

[5] Hoke CH, Nisalak A, Sangawhipa N, Jatanasen S, Laorakapongse T (1988). Protection against Japanese encephalitis by inactivated vaccines.. N Engl J Med.

[6] Xin YY, Ming ZG, Peng GY, Jian A, Min LH (1988). Safety of a live-attenuated Japanese encephalitis virus vaccine (SA14-14-2) for children.. Am J Trop Med Hyg.

[7] Ku CC, King CC, Lin CY, Hsu HC, Chen LY (1994). Homologous and heterologous neutralization antibody responses after immunization with Japanese encephalitis vaccine among Taiwan children.. J Med Virol.

[8] Olsen SJ, Supawat K, Campbell AP, Anantapreecha S, Liamsuwan S (2010). Japanese encephalitis virus remains an important cause of encephalitis in Thailand.. Int J Infect Dis.

[9] Mackenzie JS, Gubler DJ, Petersen LR (2004). Emerging flaviviruses: the spread and resurgence of Japanese encephalitis, West Nile and dengue viruses.. Nat Med.

[10] Ghosh D, Basu A (2009). Japanese encephalitis-a pathological and clinical perspective.. PLoS Negl Trop Dis.

[11] Chen CJ, Ou YC, Lin SY, Raung SL, Liao SL (2010). Glial activation involvement in neuronal death by Japanese encephalitis virus infection.. J Gen Virol.

[12] Ghoshal A, Das S, Ghosh S, Mishra MK, Sharma V (2007). Proinflammatory mediators released by activated microglia induces neuronal death in Japanese encephalitis.. Glia.

[13] Bakker AB, Baker E, Sutherland GR, Phillips JH, Lanier LL (1999). Myeloid DAP12-associating lectin (MDL)-1 is a cell surface receptor involved in the activation of myeloid cells.. Proc Natl Acad Sci U S A.

[14] Lanier LL, Corliss BC, Wu J, Leong C, Phillips JH (1998). Immunoreceptor DAP12 bearing a tyrosine-based activation motif is involved in activating NK cells.. Nature.

[15] Chen ST, Lin YL, Huang MT, Wu MF, Cheng SC (2008). CLEC5A is critical for dengue-virus-induced lethal disease.. Nature.

[16] Miller JL, deWet BJ, Martinez-Pomares L, Radcliffe CM, Dwek RA (2008). The mannose receptor mediates dengue virus infection of macrophages.. PLoS Pathog.

[17] Joyce-Shaikh B, Bigler ME, Chao CC, Murphy EE, Blumenschein WM (2010). Myeloid DAP12-associating lectin (MDL)-1 regulates synovial inflammation and bone erosion associated with autoimmune arthritis.. J Exp Med.

[18] Aoki N, Kimura Y, Kimura S, Nagato T, Azumi M (2009). Expression and functional role of MDL-1 (CLEC5A) in mouse myeloid lineage cells.. J Leukoc Biol.

[19] Winter PM, Dung NM, Loan HT, Kneen R, Wills B (2004). Proinflammatory cytokines and chemokines in humans with Japanese encephalitis.. J Infect Dis.

[20] Ford AL, Goodsall AL, Hickey WF, Sedgwick JD (1995). Normal adult ramified microglia separated from other central nervous system macrophages by flow cytometric sorting. Phenotypic differences defined and direct ex vivo antigen presentation to myelin basic protein-reactive CD4+ T cells compared.. J Immunol.

[21] German AC, Myint KS, Mai NT, Pomeroy I, Phu NH (2006). A preliminary neuropathological study of Japanese encephalitis in humans and a mouse model.. Trans R Soc Trop Med Hyg.

[22] Liang JJ, Liao CL, Liao JT, Lee YL, Lin YL (2009). A Japanese encephalitis virus vaccine candidate strain is attenuated by decreasing its interferon antagonistic ability.. Vaccine.

[23] Andersson LM, Hagberg L, Fuchs D, Svennerholm B, Gisslen M (2001). Increased blood-brain barrier permeability in neuro-asymptomatic HIV-1-infected individuals–correlation with cerebrospinal fluid HIV-1 RNA and neopterin levels.. J Neurovirol.

[24] Mishra MK, Dutta K, Saheb SK, Basu A (2009). Understanding the molecular mechanism of blood-brain barrier damage in an experimental model of Japanese encephalitis: correlation with minocycline administration as a therapeutic agent.. Neurochem Int.

[25] Huber JD, Egleton RD, Davis TP (2001). Molecular physiology and pathophysiology of tight junctions in the blood-brain barrier.. Trends Neurosci.

[26] Getts DR, Terry RL, Getts MT, Muller M, Rana S (2008). Ly6c+ “inflammatory monocytes” are microglial precursors recruited in a pathogenic manner in West Nile virus encephalitis.. J Exp Med.

[27] Geissmann F, Jung S, Littman DR (2003). Blood monocytes consist of two principal subsets with distinct migratory properties.. Immunity.

[28] Cheung R, Shen F, Phillips JH, McGeachy MJ, Cua DJ (2011). Activation of MDL-1 (CLEC5A) on immature myeloid cells triggers lethal shock in mice.. J Clin Invest.

[29] Felderhoff-Mueser U, Schmidt OI, Oberholzer A, Buhrer C, Stahel PF (2005). IL-18: a key player in neuroinflammation and neurodegeneration?. Trends Neurosci.

[30] Gruol DL, Nelson TE (1997). Physiological and pathological roles of interleukin-6 in the central nervous system.. Mol Neurobiol.

[31] Leonard EJ, Yoshimura T (1990). Human monocyte chemoattractant protein-1 (MCP-1).. Immunol Today.

[32] Block ML, Zecca L, Hong JS (2007). Microglia-mediated neurotoxicity: uncovering the molecular mechanisms.. Nat Rev Neurosci.

[33] Allan SM, Rothwell NJ (2001). Cytokines and acute neurodegeneration.. Nat Rev Neurosci.

[34] Abraham M, Shapiro S, Lahat N, Miller A (2002). The role of IL-18 and IL-12 in the modulation of matrix metalloproteinases and their tissue inhibitors in monocytic cells.. Int Immunol.

[35] Wheeler RD, Brough D, Le Feuvre RA, Takeda K, Iwakura Y (2003). Interleukin-18 induces expression and release of cytokines from murine glial cells: interactions with interleukin-1 beta.. J Neurochem.

[36] Gupta AK, Lad VJ, Koshy AA (2009). Early death of Japanese encephalitis virus-infected mice administered a neutralizing cross-reactive monoclonal antibody against glycoprotein E.. Acta Virol.

[37] Watson AA, Lebedev AA, Hall BA, Fenton-May AE, Vagin AA (2011). Structural flexibility of the macrophage dengue virus receptor CLEC5A: Implications for ligand binding and signaling.. J Biol Chem.

[38] Hsu TL, Cheng SC, Yang WB, Chin SW, Chen BH (2009). Profiling carbohydrate-receptor interaction with recombinant innate immunity receptor-Fc fusion proteins.. J Biol Chem.

[39] Lin YL, Liao CL, Yeh CT, Chang CH, Huang YL (1996). A highly attenuated strain of Japanese encephalitis virus induces a protective immune response in mice.. Virus Res.

[40] Saura J, Tusell JM, Serratosa J (2003). High-yield isolation of murine microglia by mild trypsinization.. Glia.

[41] Cardona AE, Huang D, Sasse ME, Ransohoff RM (2006). Isolation of murine microglial cells for RNA analysis or flow cytometry.. Nat Protoc.

[42] Ohtsuki S, Yamaguchi H, Katsukura Y, Asashima T, Terasaki T (2008). mRNA expression levels of tight junction protein genes in mouse brain capillary endothelial cells highly purified by magnetic cell sorting.. J Neurochem.

[43] Liang JJ, Yu CY, Liao CL, Lin YL (2011). Vimentin binding is critical for infection by the virulent strain of Japanese encephalitis virus.. Cell Microbiol.

[44] Durbin JE, Hackenmiller R, Simon MC, Levy DE (1996). Targeted disruption of the mouse Stat1 gene results in compromised innate immunity to viral disease.. Cell.

[45] Becker HM, Chen M, Hay JB, Cybulsky MI (2004). Tracking of leukocyte recruitment into tissues of mice by in situ labeling of blood cells with the fluorescent dye CFDA SE.. J Immunol Methods.

