# Supplementary figures and images for: CLEC5A Regulates Japanese Encephalitis Virus-Induced Neuroinflammation and Lethality
Source: PLoS Pathog. 2012 Apr 19;8(4):e1002655. doi: 10.1371/journal.ppat.1002655 (PMC3334897; doi:10.1371/journal.ppat.1002655)

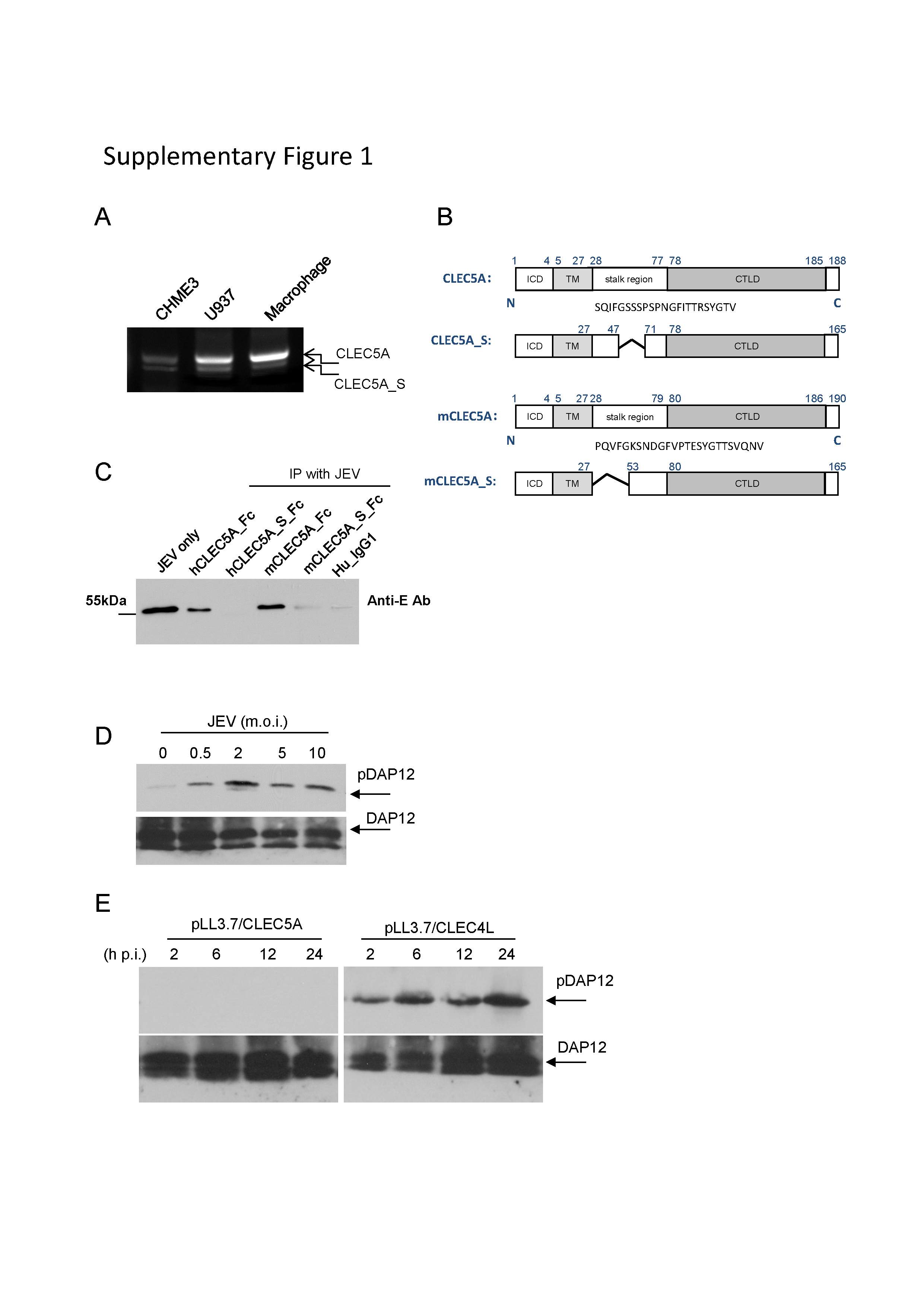

Supplement: Figure S1 — JEV interacts with CLEC5A and induces DAP12 phosphorylation via CLEC5A. (A) Expression of human CLEC5A and an alternatively spliced variant (CLEC5A_S) in human microglial cell line (CHME3), monocytic cell line (U937) and CD14+-monocyte derived macrophages (MoM) was detected by RT-PCR. (B) Schematic representation of human and murine CLEC5A. Sequence analysis of PCR products revealed that human CLEC5A_S lacks 23 amino acids in the stalk region (aa 48–70), while mCLEC5A_S lacks 25 amino acids (aa 28–52) located in stalk regions due to alternative splicing. (C) JEV–CLEC5A.Fc complexes were immunoprecipitated with protein A–Sepharose and detected by anti-JEV envelope protein mAb. (D) JEV-induced DAP12 phosphorylation (1 h post infection) in human macrophages was determined by western blotting. (E) Effects of shRNAs (pLL3.7 backbone) on inhibition of JEV-mediated DAP12 phosphorylation were determined by western blotting (h.p.i., hours post infection). The pLL3.7/CLEC4L was used as a control shRNA. (TIF) [file ppat.1002655.s001.tif]

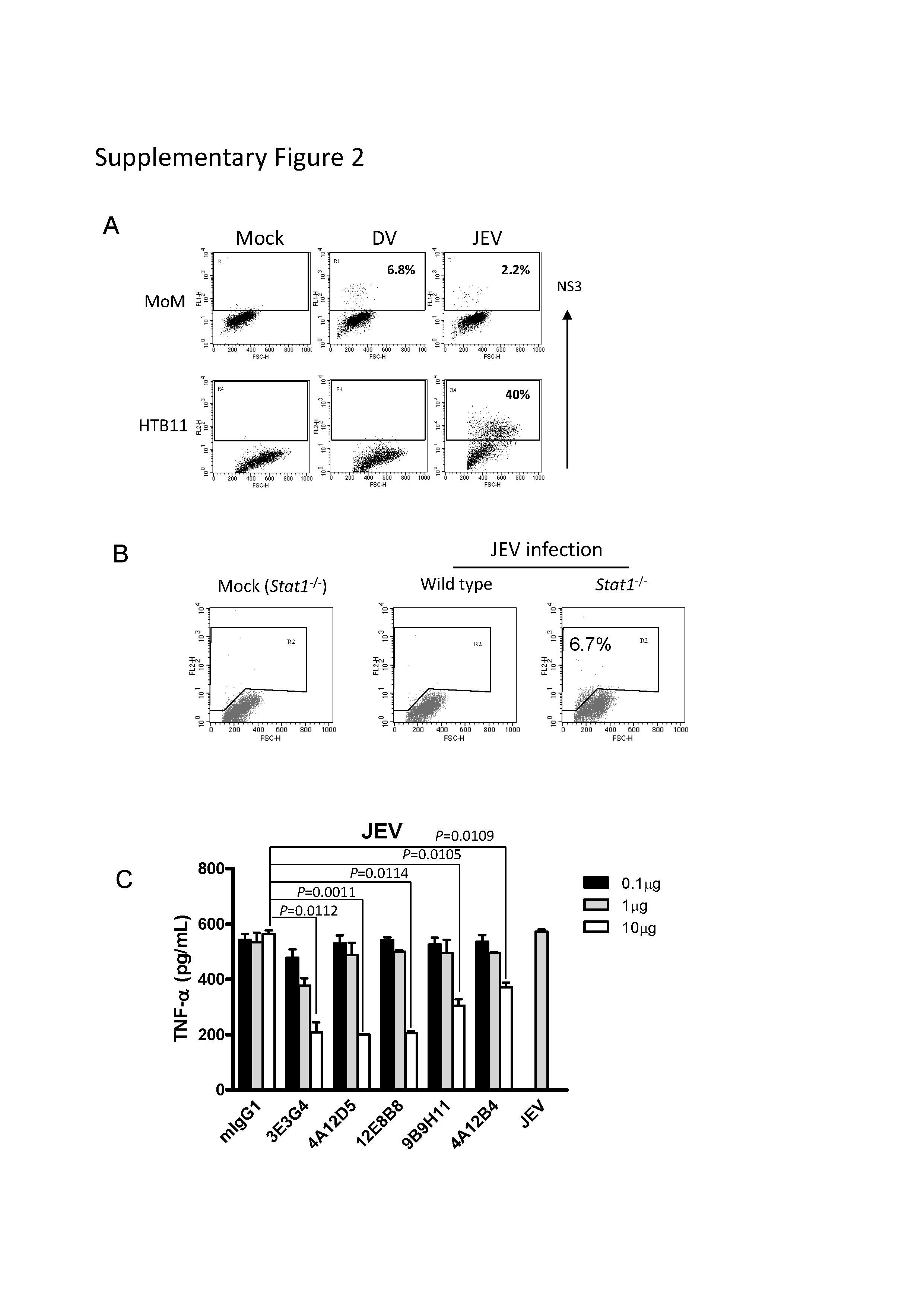

Supplement: Figure S2 — JEV replicates in macrophages and induces cytokine release. (A) Human MoM and HTB11, (a human neuroblastoma cell line) infected with DV or JEV (m.o.i. = 5) were subjected to flow cytometry analysis at 48 h post infection using an antibody to nonstructural protein 3 (NS3) to detect viral antigens. (B) Murine bone marrow-derived macrophages (BMDM) from wild type and Stat1 −/− mice were infected with JEV (m.o.i. = 5), followed by anti JEV-NS3 mAb staining and FACS analysis. (C) BMDM from Stat1 −/− mice were infected with DV JEV (m.o.i. = 5) in the absence or presence of different doses of anti-CLEC5A mAbs, and supernatants were harvested at 48 h post infection for cytokine determination by ELISA. Data were collected and expressed as mean ± s.e.m. from at least three independent experiments. Two-tailed Student's t-tests were performed. (TIF) [file ppat.1002655.s002.tif]

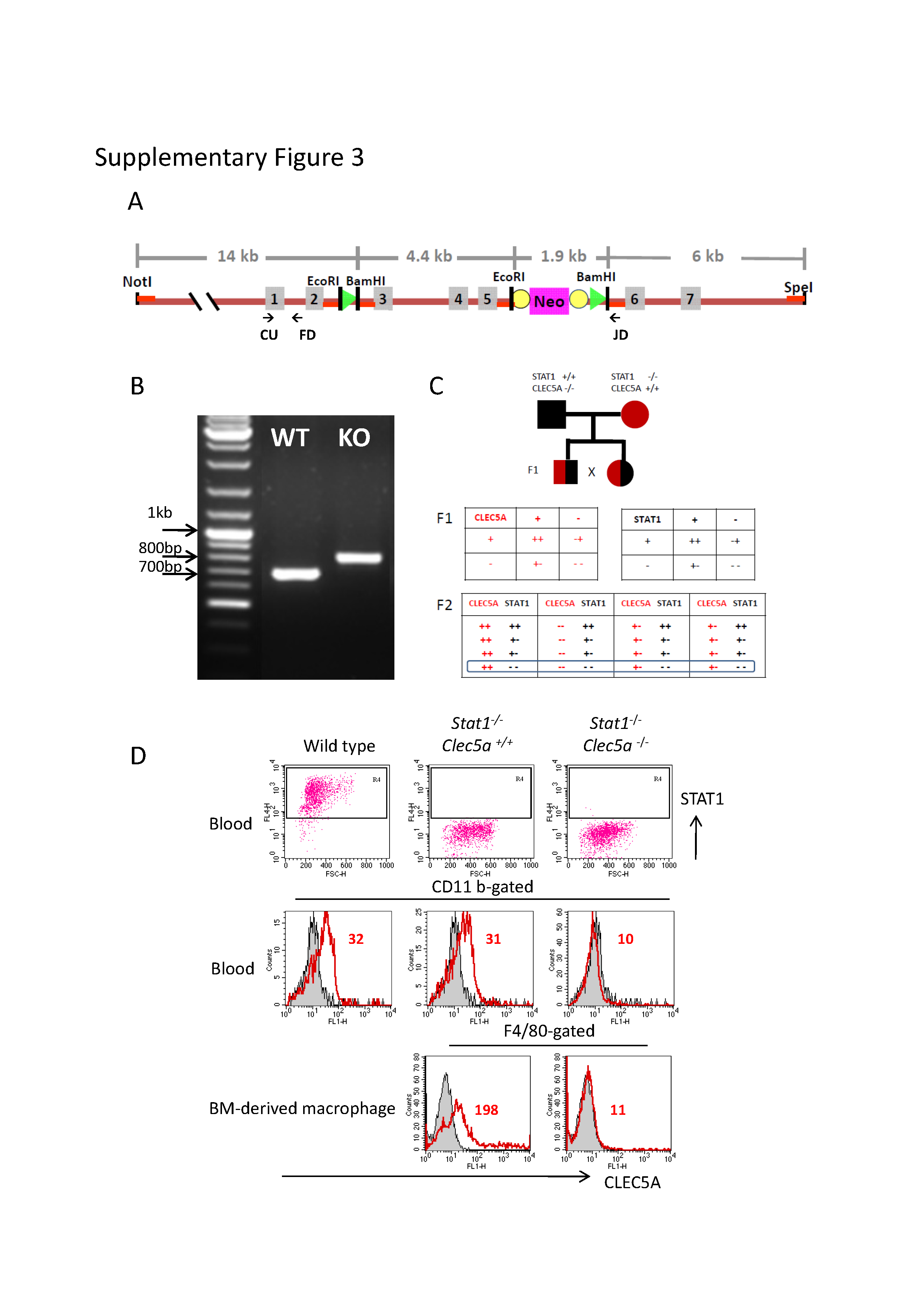

Supplement: Figure S3 — Targeting strategy for generation of CLEC5A KO mice and CLEC5A and STAT1 double KO mice. (A) Targeting vector for generation of Clec5a −/− mice. A neomycin resistance gene cassette (NEO) was introduced into targeting vector for positive selection; and two loxP sequences (green triangles) flanking exons 3 to 5 allow the removal of CLEC5A exon 3–5 using a Cre-loxP excision system. Locations of PCR primers used for genotyping are shown under targeting vector. (B) Genotyping by PCR using CU+FD and CU+JD primer sets for wild type and Clec5a −/− mouse, respectively. (C) Double KO mice were produced by mating Clec5a+/+ Stat1−/− and Clec5a−/− Stat1+/+ mice, and the F1 offspring were further interbred to generate F2 offspring. (D) Determination of CLEC5A and STAT1 expression in peripheral blood cells by flow cytometry. (TIF) [file ppat.1002655.s003.tif]

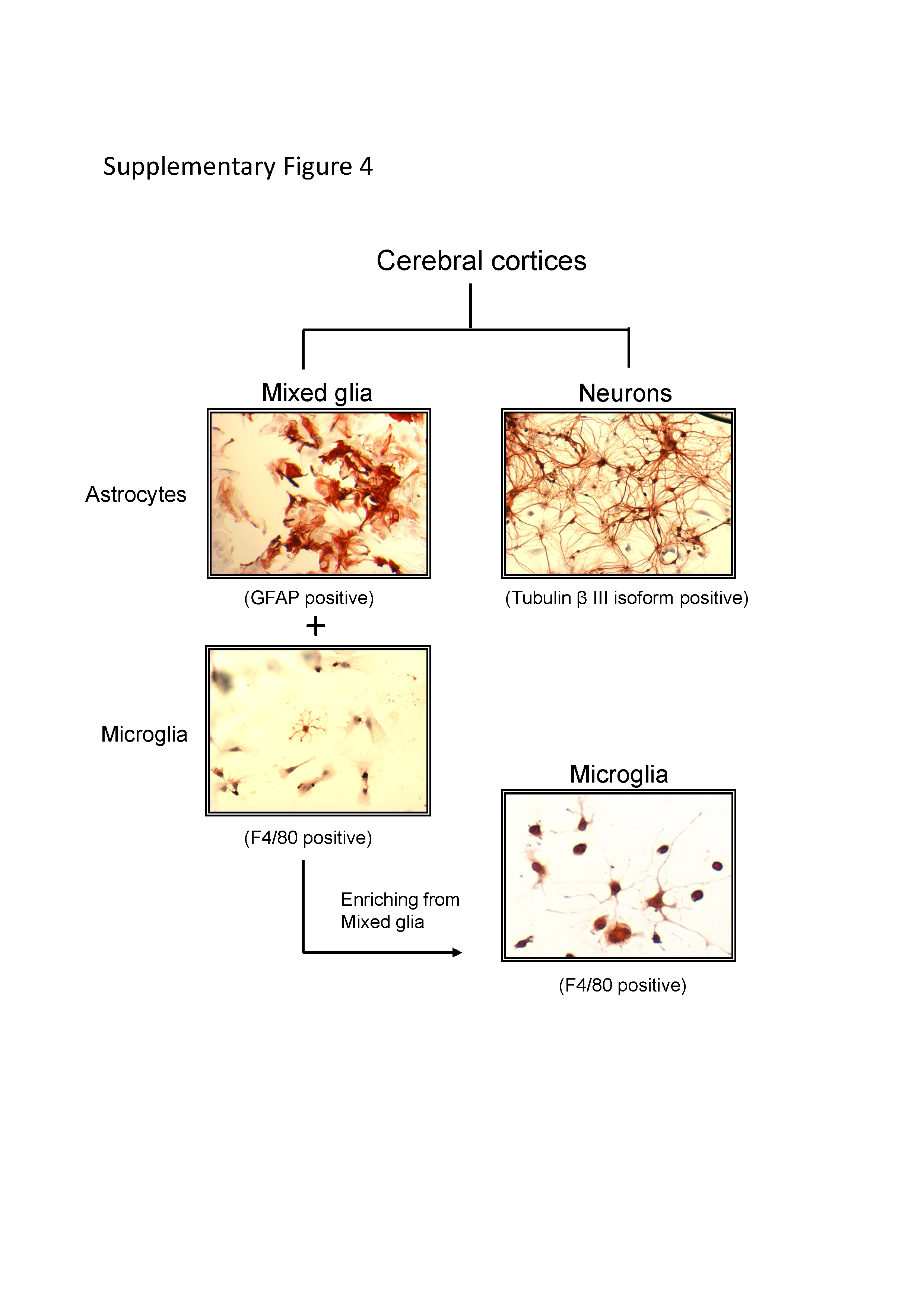

Supplement: Figure S4 — Flow chart for isolation of glia cells and mixed glia fractions from cerebral cortices. Neurons and mixed glia were prepared from the cerebral cortices of neonatal STAT1 −/− mice, and differentiated in neurobasal medium supplemented with B27 (Life Technologies) and DMEM/F12 (Life Technologies) supplemented with 10% (v/v) FCS, respectively. Microglia were further enriched from differentiated mixed glial cell cultures. Neurons, astrocytes and microglia were characterized by staining with antibodies to tubulin β III isoform, glial fibrillary acidic protein (GFAP), or F4/80, respectively. Mixed glial cultures contained ∼85% astrocytes and ∼10% microglia. The purity of neurons and microglia was >95%. (TIF) [file ppat.1002655.s004.tif]

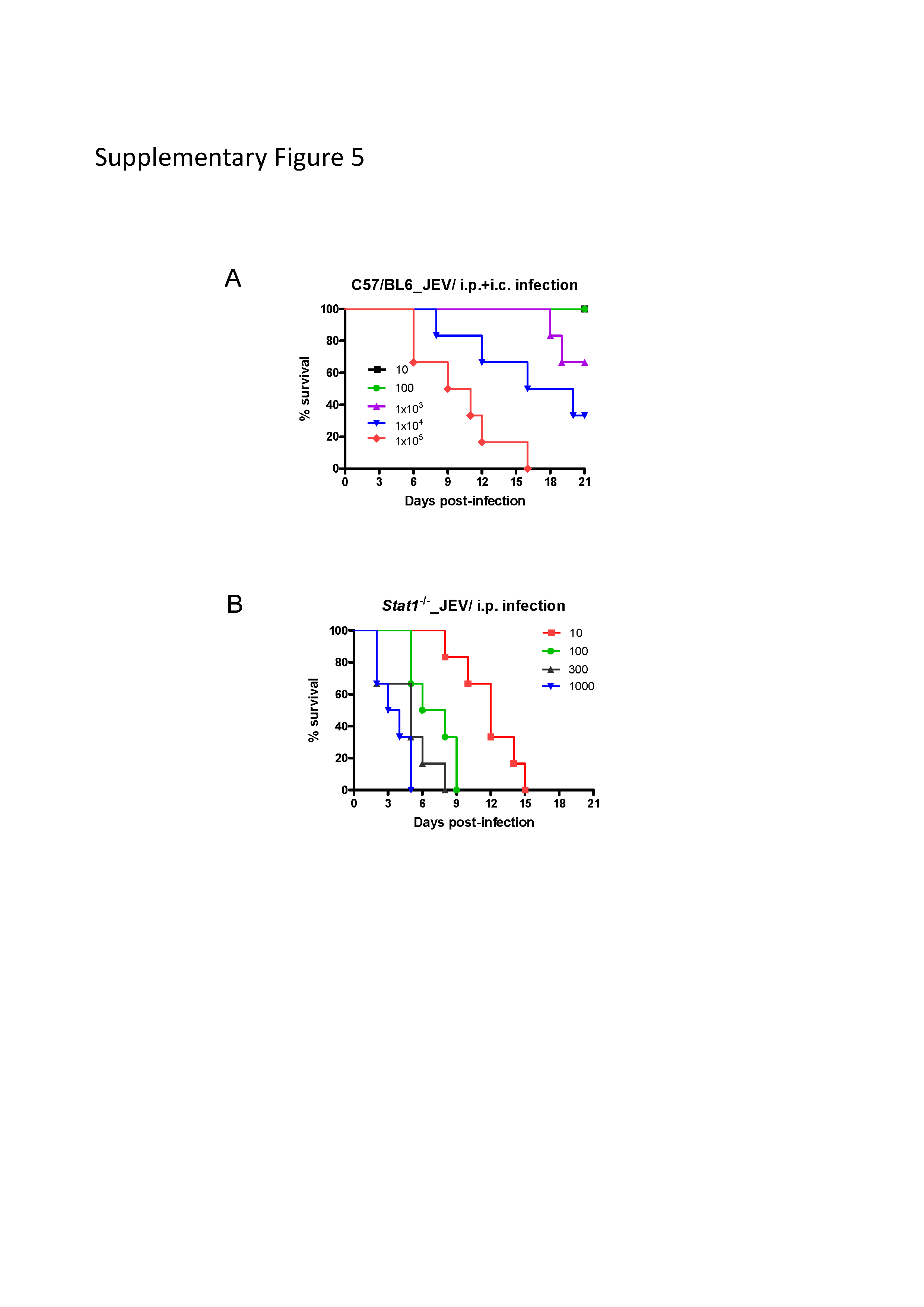

Supplement: Figure S5 — Murine models for JEV infection. (A) Wild-type C57BL/6 mice (n = 10 per group) were challenged with various doses of JEV (pfu) via an intraperitoneal route with intracranial injection of 30 µL PBS simultaneously (i.p.+i.c.). (B) STAT1-deficient mice were intraperitoneally infected with a range of doses of JEV (n = 6 per group). All mice were monitored daily for 21 days and outcomes are shown as Kaplan–Meier survival curves with log rank test. (TIF) [file ppat.1002655.s005.tif]

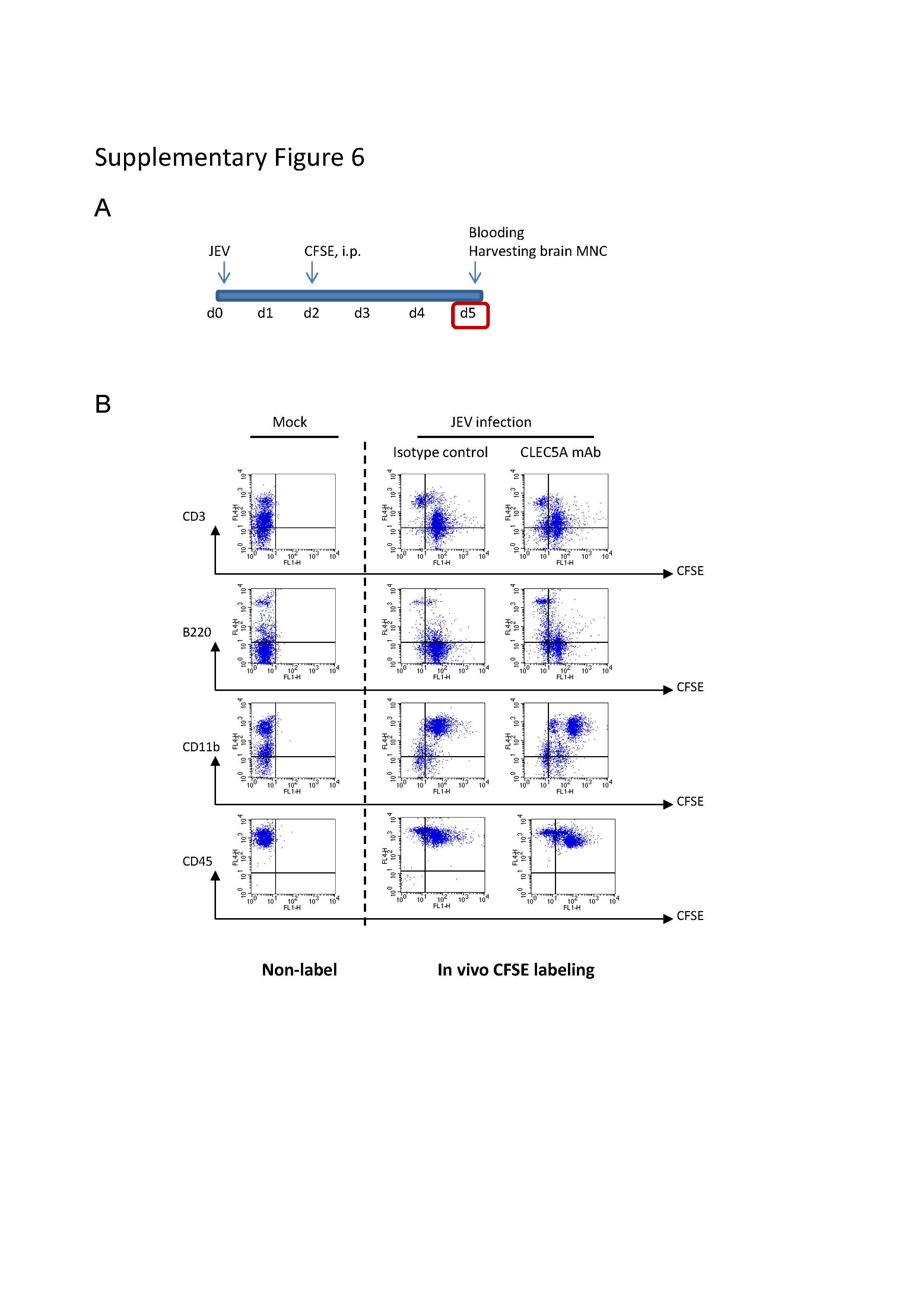

Supplement: Figure S6 — In situ labeling of peripheral leukocytes with CFSE in JEV-infected mice. (A) Schematic representation of the procedure for in situ labeling with CFSE fluorescence dye in JEV-infected mice. (B) Validation of CFSE labeling efficiency by analyzing fluorescence intensity in peripheral blood leukocytes from JEV-infected mice at 72 hr after CFSE injection (day 5 after JEV infection). (TIF) [file ppat.1002655.s006.tif]

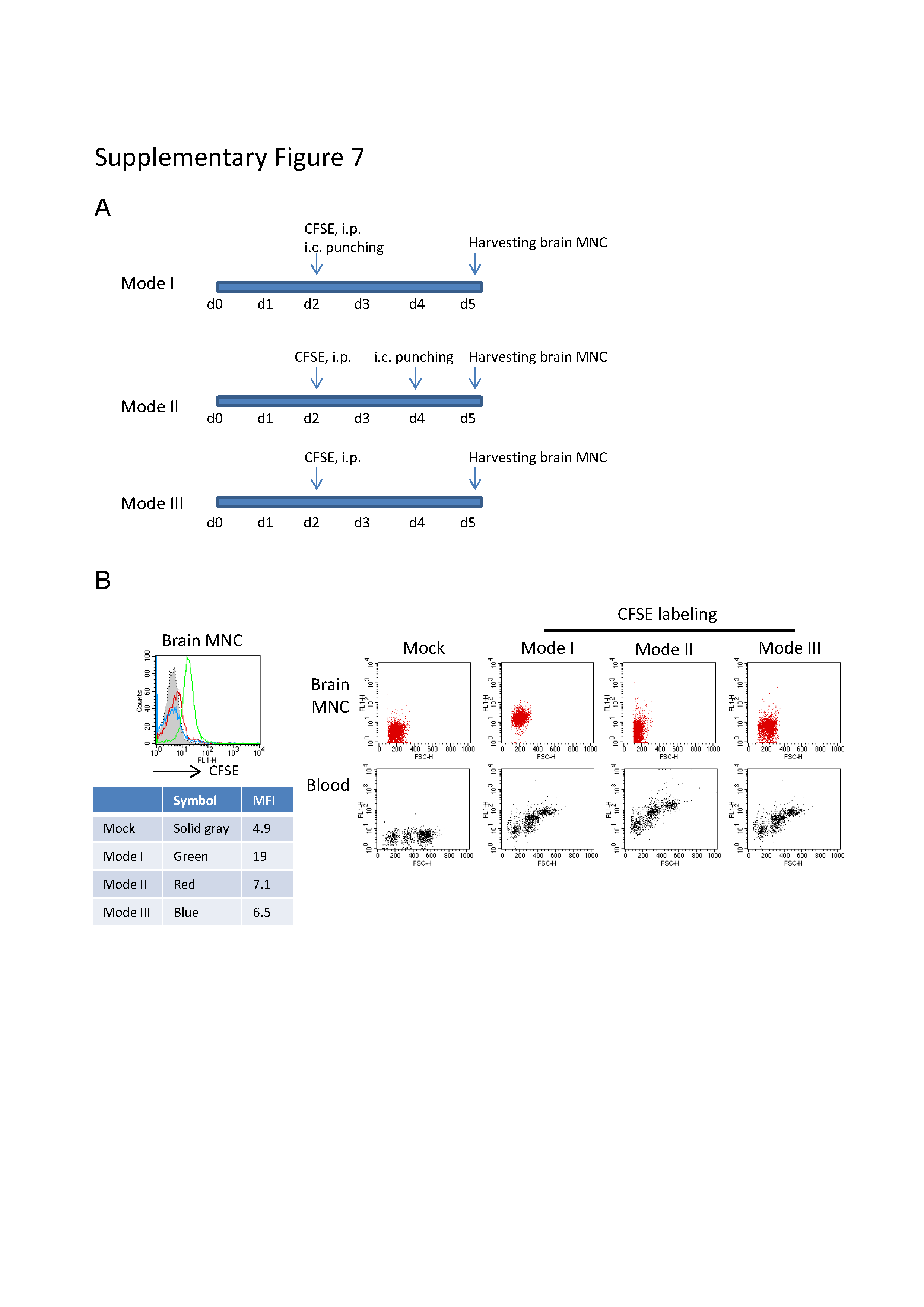

Supplement: Figure S7 — CFSE distribution into CNS after intracranial puncture. (A) Three modes to evaluate the distribution of CFSE after i.p. injection to mice with or without intracranial i.c. puncture. (B) Analysis of CFSE fluorescence in the MNCs isolated from brain MNCs using Percoll-gradient centrifugation. MFI of each group was shown in left panel. MFI: mean fluorescence intensity. (TIF) [file ppat.1002655.s007.tif]

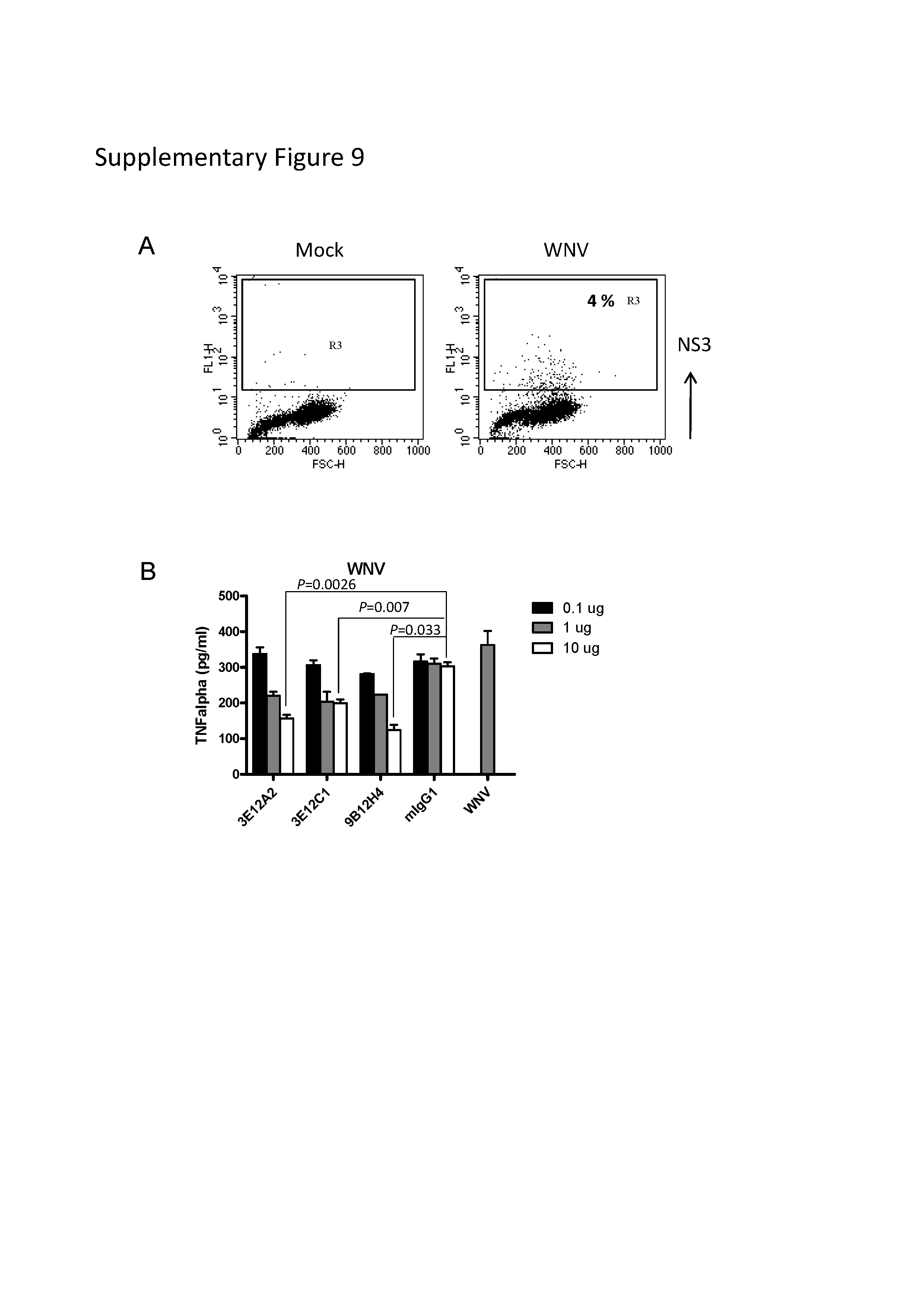

Supplement: Figure S9 — WNV replicates in human macrophages and antagonistic anti-CLEC5A mAbs inhibit WNV-induced macrophage activation. (A) Human CD14+-monocyte derived macrophages (MoM) infected with WNV (m.o.i. = 5) were subjected to flow cytometry analysis at 48 hr post infection using anti-NS3 antibody to determine WNV replication. (B) Dose-dependent inhibition of cytokine release from WNV-infected MoM by anti-CLEC5A mAbs (clones: 3E12A2, 3E1C1 and 9B12H4) determined by ELISA at 48 hr post infection. mIgG1 acts as an isotype matched control. Data were collected from at least three different donors and expressed as mean ± s.d. Two-tailed Student's t-tests were performed. (TIF) [file ppat.1002655.s009.tif]

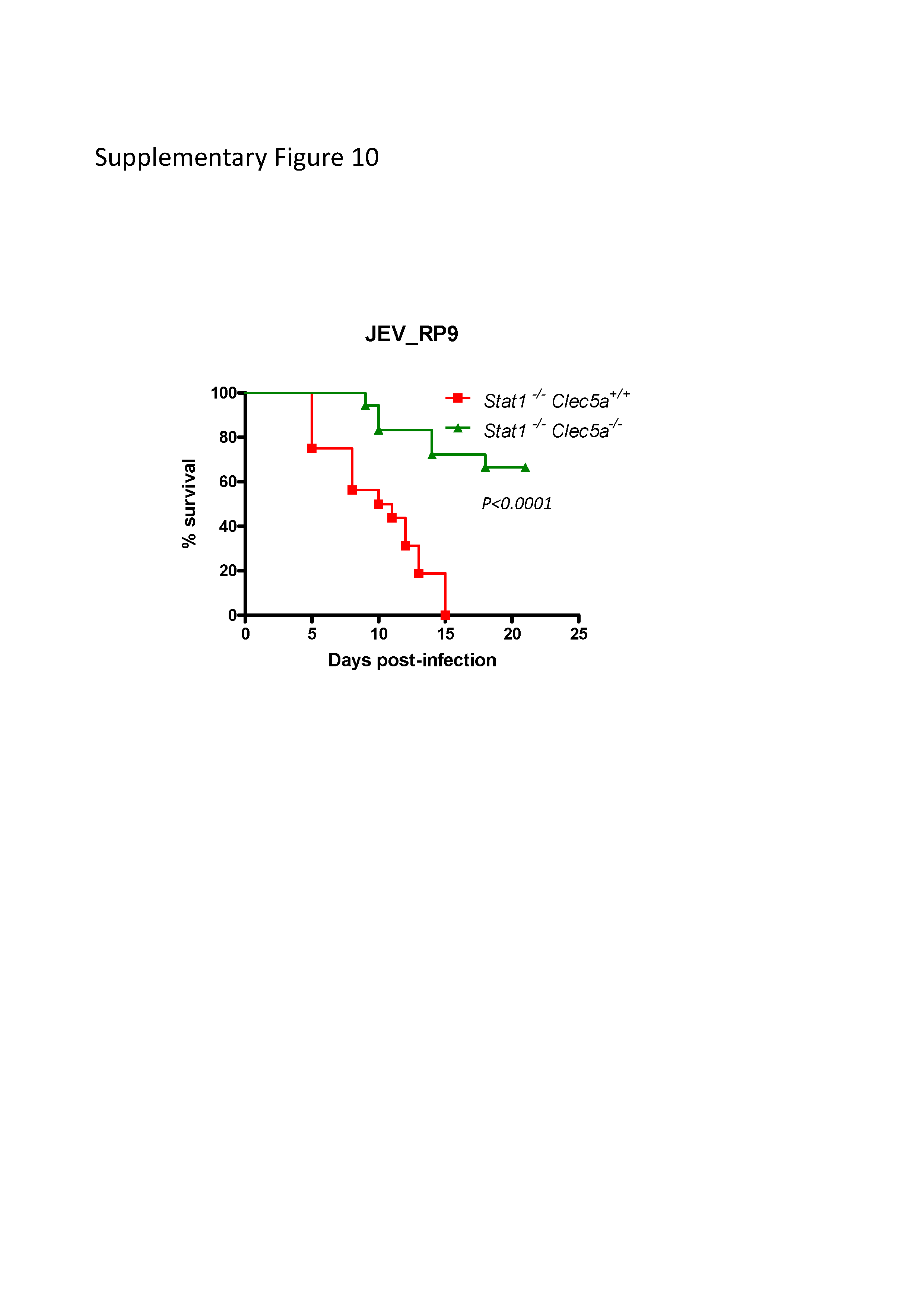

Supplement: Figure S10 — Stat1 −/ − Clec5a −/− mice are resistant to JEV-infection. Survival of both the Stat1 −/− Clec5a +/+ and Stat1 −/− Clec5a −/− mice (8–10 weeks) was monitored for 21 days after i.p. inoculation of JEV (100 pfu/mice); data were collected from four independent experiments and are shown as Kaplan–Meier survival curves with log rank test; n = 20 for each group. (TIF) [file ppat.1002655.s010.tif]
